# Breaking steric hindrance restriction: compressed metal bonds realizing deep-level orbital delocalization

**DOI:** 10.1093/nsr/nwag388

**Published:** 2026-07-06

**Authors:** Hanghao Ying, Liuxin Xu, Xia Zhong, Yi Liu, Xiaodong Zhang, Yi Xie

**Affiliations:** Hefei National Research Center for Physical Sciences at the Microscale, University of Science and Technology of China, Hefei 230026, China; Hefei National Research Center for Physical Sciences at the Microscale, University of Science and Technology of China, Hefei 230026, China; Hefei National Research Center for Physical Sciences at the Microscale, University of Science and Technology of China, Hefei 230026, China; Hefei National Research Center for Physical Sciences at the Microscale, University of Science and Technology of China, Hefei 230026, China; Hefei National Research Center for Physical Sciences at the Microscale, University of Science and Technology of China, Hefei 230026, China; Hefei National Research Center for Physical Sciences at the Microscale, University of Science and Technology of China, Hefei 230026, China; State Key Laboratory of Precision and Intelligent Chemistry, University of Science and Technology of China, Hefei 230026, China

**Keywords:** deep-level delocalization, compressed metal bonds, steric hindrance effect, photocatalysis

## Abstract

The introduction of large functional groups is a well-established strategy to enhance the stability and value of chemicals. However, accompanying steric hindrance effects can significantly impede the reaction progress, presenting a critical dilemma in synthetic design. Herein, a vacancy-constrained strategy was proposed for breaking steric hindrance restriction by realizing the deep-level delocalization of a catalyst with compressed metal bonds. In detail, the delocalized deep-level orbit exhibits strong penetrating capability that overcomes the steric hindrance effect of large functional groups by enhancing the charge-transfer process between the active sites and substrates. By taking bismuth atoms supported on defective CeO_2_ as an example, an ‘O_4_-Bi-Bi-O_4_’ ensemble was constructed by confining two adjacent Bi atoms in the vacancy of CeO_2_ nanosheets, where the Bi–Bi bond was strongly constrained from 3.10 to 3.05 Å. Such a functional Bi_2_/CeO_2_ catalyst exhibits enhanced photocatalytic efficiency in overcoming the steric hindrance from large functional groups, which is three times and two times higher than that of Bi single-atom catalyst and Bi cluster catalyst, respectively. This work proposes an applicable way of overcoming steric hindrance effects in the synthesis of high-value-added chemicals.

## Teaser

By compressing metal bonds, a novel catalyst design effectively overcomes the spatial crowding of large molecular groups, significantly accelerating the efficient synthesis of high-value-added chemicals.

## INTRODUCTION

The strategic introduction of large functional groups has long been a fundamental approach in high-value-added chemical synthesis, serving as a powerful tool to enhance product stability and enable precise engineering of functional properties for specific applications. For example, in pharmaceutical design, such bulky substituents are routinely employed to fine-tune metabolic stability, while in petrochemical applications they optimize material performance [1–6]. However, this strategy inevitably introduces significant steric constraints in synthetic processes, which can lead to obstruction of reactive sites, significant kinetic retardation, or even complete prevention of desired transformations [7–9]. Taking the photocatalytic epoxide cycloaddition reaction as an example, the product of styrene carbonate with the large phenyl group (–C_6_H_5_) exhibits superior stability and low viscosity when employed as electrolytes in secondary batteries in comparison to propylene carbonate with the methyl group (–CH_3_), with the value of styrene carbonate being ∼100 times higher than that of propylene carbonate [10]. However, the high steric hindrance of styrene oxide severely restricts its photocatalytic conversion rate, because the poor electron-withdrawing ability of the phenyl group weakens the adsorption of styrene oxide on the catalyst surface. Furthermore, it is difficult for the photogenerated electrons to penetrate the *π*-bond electron clouds of the phenyl group and activate the three-membered ring of styrene oxide [11–13]. These challenges underscore the necessity for developing advanced heterogeneous catalysts with delocalized electron states capable of overcoming steric hindrance effects from reactant molecules.

**Figure 1.**
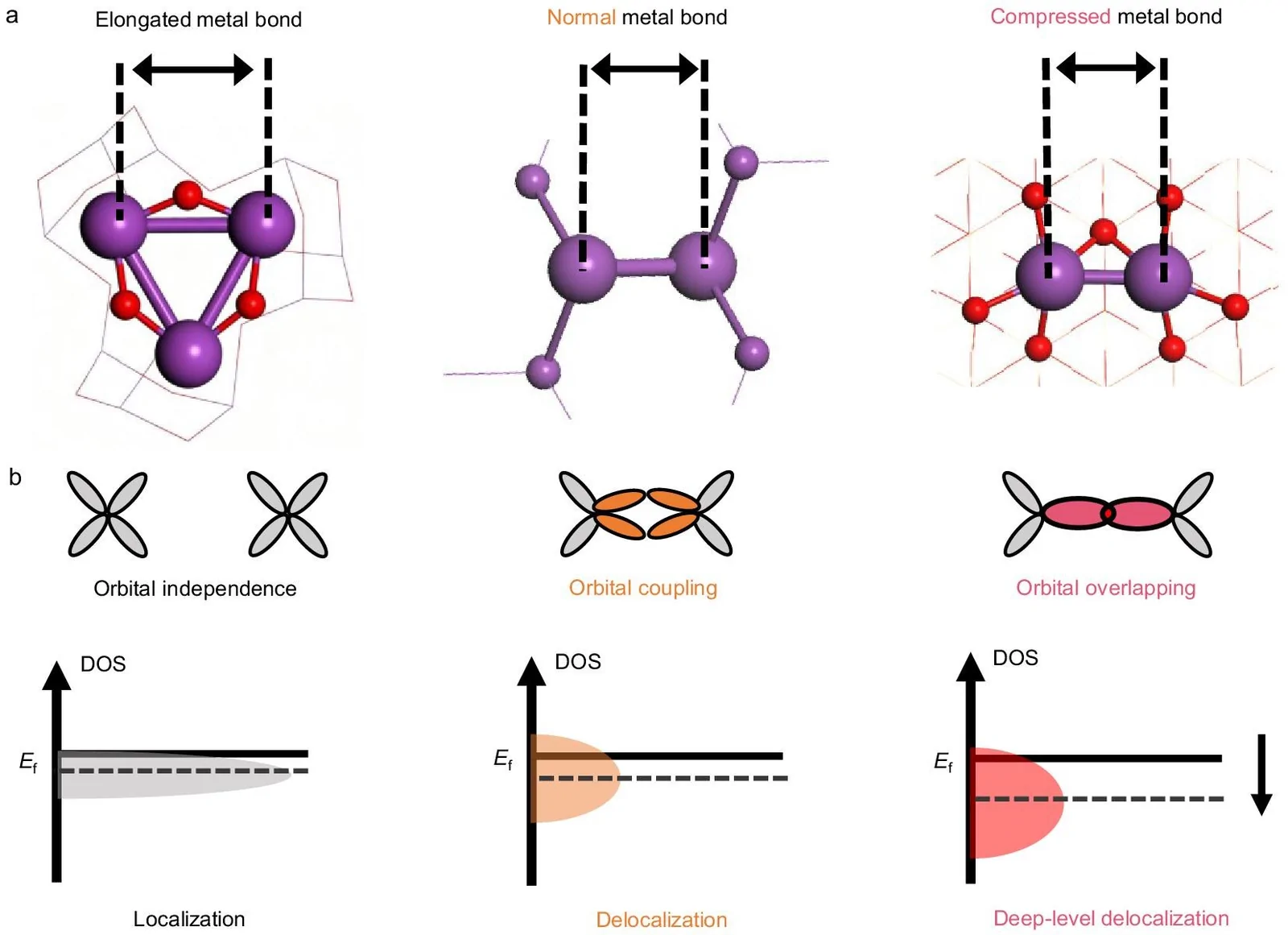
Schematic diagram of the relationship between the length of metal bonds and electronic states of catalysts. (a) Different lengths of metal bonds in various models for metallic oxide (left), metal (middle) and M_2_/CeO_2_ model (right), respectively, where the purple balls represent metal atoms and red balls represent O atoms. (b) Schematic representation of DOS showing that the compressed metal bonds lead to the orbital overlapping and deep-level delocalization of electron states.

In recent years, supported metal catalysts have attracted great attention due to their distinctive interface effect and easy-tunable electronic structure, such as single-atom catalysts and supported metal cluster catalysts [14–17]. The metal-support interaction in these systems would induce strong covalent coupling between the outer orbitals of metal atoms and support atoms, realizing enhanced reactivity of the active sites. However, conventional supported metal catalysts fail to provide delocalized electrons with sufficient energy to penetrate the sterically hindered electron clouds from large functional groups in high-value-added chemical synthesis. To address this limitation, modulating the localized microenvironments of supported metal catalysts through metal orbital overlapping has emerged as a promising strategy [18,19]. Compared with the metal orbitals in metallic oxide and pure metal, such orbital overlapping could promote the delocalization of electron states into deep levels (Fig. 1), effectively overcoming the constraints imposed by steric hindrance effects.

Herein, we propose a vacancy-constrained strategy by compressing metal bonds in supported metal catalysts to overcome the steric hindrance effect of molecules within large functional groups. Taking bismuth atoms supported on defective CeO_2_ as an example (denoted as Bi*_x_*/CeO_2_), two adjacent Bi atoms are constrained at the vacancy on CeO_2_ nanosheets to form a compressed Bi–Bi bond in an ‘O_4_-Bi-Bi-O_4_’ ensemble. Theoretical studies reveal that the shortened Bi–Bi bonds result in the overlap of the Bi-*p* orbital and coupling of the electronic distribution, thus inducing the delocalization of Bi electron states into deep levels. This deep-level orbital delocalization provides an exceptional electron penetrating capability to break the steric hindrance of electronic clouds from the large functional groups. Consequently, this unique electronic configuration significantly enhances the adsorption and activation of organic molecules with large functional groups, providing a promising pathway for catalytic transformations of sterically hindered substrates.

## RESULTS AND DISCUSSION

**Figure 2.**
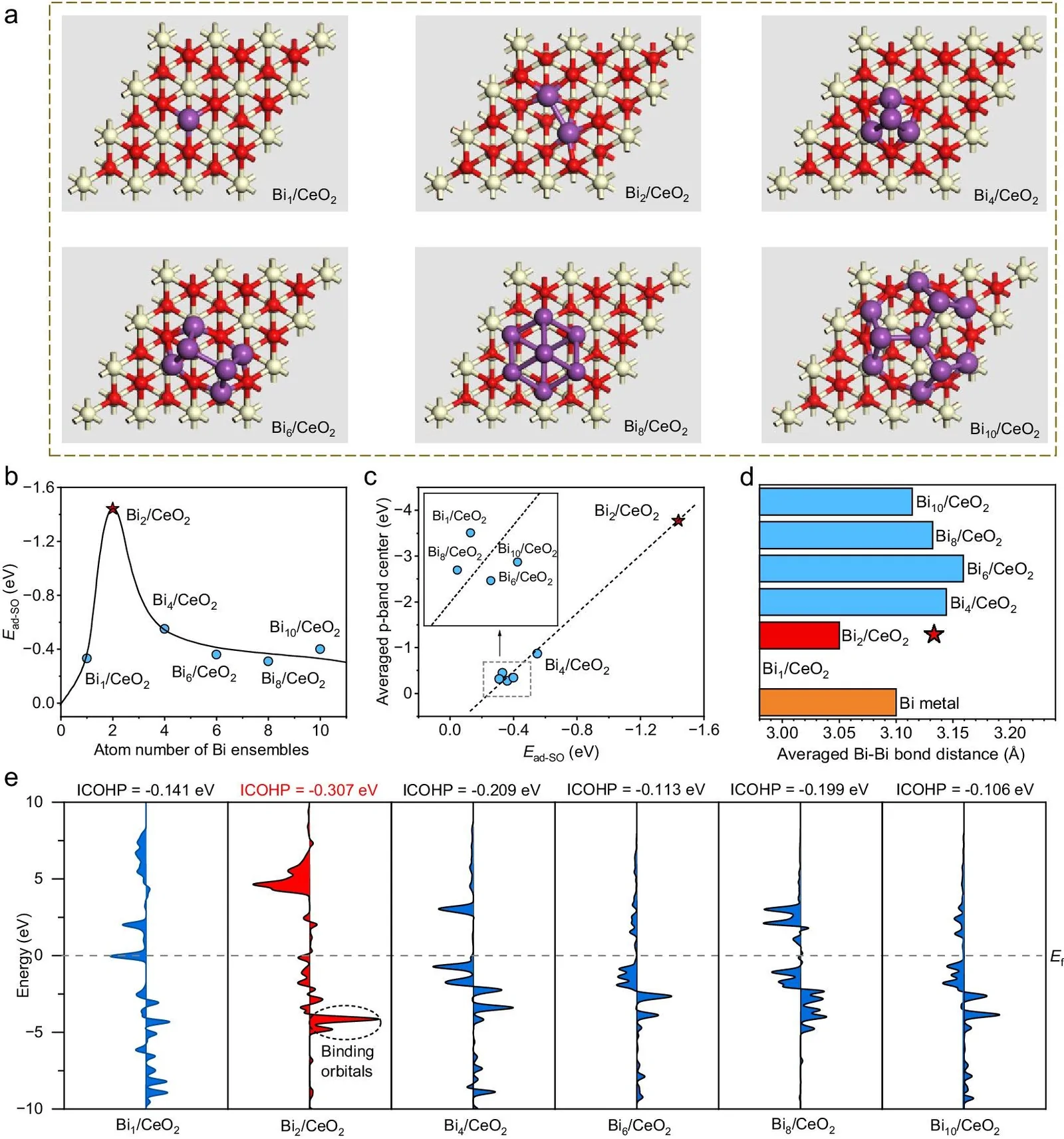
Computational screening of Bi*_x_*/CeO_2_ with various structure models. (a) Different models employed in DFT calculations. (b) Adsorption energy of styrene oxide for Bi*_x_*/CeO_2_. (c) Relationship between averaged *p*-band center and the adsorption energy of styrene oxide. (d) Averaged Bi–Bi bond in Bi*_x_*/CeO_2_. (e) Crystal Orbital Hamilton Population (COHP) analysis of the Bi atom in Bi*_x_*/CeO_2_ and the O atom in styrene oxide.

To systematically investigate the activation capability of our catalyst system for sterically hindered molecules, we constructed an ‘O_4_-Bi-Bi-O_4_’ ensemble model featuring two Bi atoms confined in a CeO_2_ vacancy, along with comparative models of various Bi*_x_*/CeO_2_ (*x* = 1, 4, 6, 8, 10) for comparison (Fig. 2a). The adsorption energy of styrene oxide (*E*_ad-SO_) with a large functional group was employed as a descriptor, with more negative values indicating a strong preference for styrene oxide activation. As illustrated in Fig. 2b, the overall performance trend of the models was encapsulated in a volcano curve, wherein the Bi_2_/CeO_2_ exhibited an exceptional adsorption capacity (*E*_ad-SO_ = −1.44 eV) at the peak position. In contrast, the Bi_1_/CeO_2_ exhibited significantly weak adsorption (*E*_ad-SO_ = −0.33 eV), indicating poor reactivity towards styrene oxide activation. The same conclusion could be drawn from the Bi_4_/CeO_2_, Bi_6_/CeO_2_, Bi_8_/CeO_2_ and Bi_10_/CeO_2_ with their *E*_ad-SO_ of −0.55, −0.36, −0.31 and −0.4 eV, respectively. To further investigate the adsorption capacity of styrene oxide for the Bi_2_/CeO_2_ model, more structural information was studied after a relaxation calculation. To our surprise, in this ensemble of Bi_2_/CeO_2_, the length of the Bi–Bi bond was constrained to 3.05 Å, a value even shorter than that of the intra-layer Bi–Bi bond length of Bi metal at 3.10 Å (Fig. 2d). This bond compression facilitated significant overlap of Bi-*p* orbitals and electronic distribution coupling (Figs S1 and S2), thereby inducing the delocalization of the Bi electron states into deep levels. The delocalization of electron states was evidenced by the lowest averaged *p*-band center position (−3.77 eV) observed for Bi_2_/CeO_2_ (Fig. 2c). Furthermore, a linear correlation between the averaged *p*-band center position of Bi atoms and *E*_ad-SO_ across different models suggested that deep-level orbital delocalization strengthens orbital hybridization between Bi atoms and oxygen atoms in adsorbed styrene oxide molecules. This conclusion was further supported by Crystal Orbital Hamilton Population (COHP) analysis, which demonstrated enhanced electron density in bonding orbitals of Bi_2_/CeO_2_, as indicated by its lower COHP value compared to other configurations (Fig. 2e). These findings suggest that Bi_2_/CeO_2_ would be the optimal candidate for overcoming the steric hindrance effect of large functional groups in photocatalysis.

**Figure 3.**
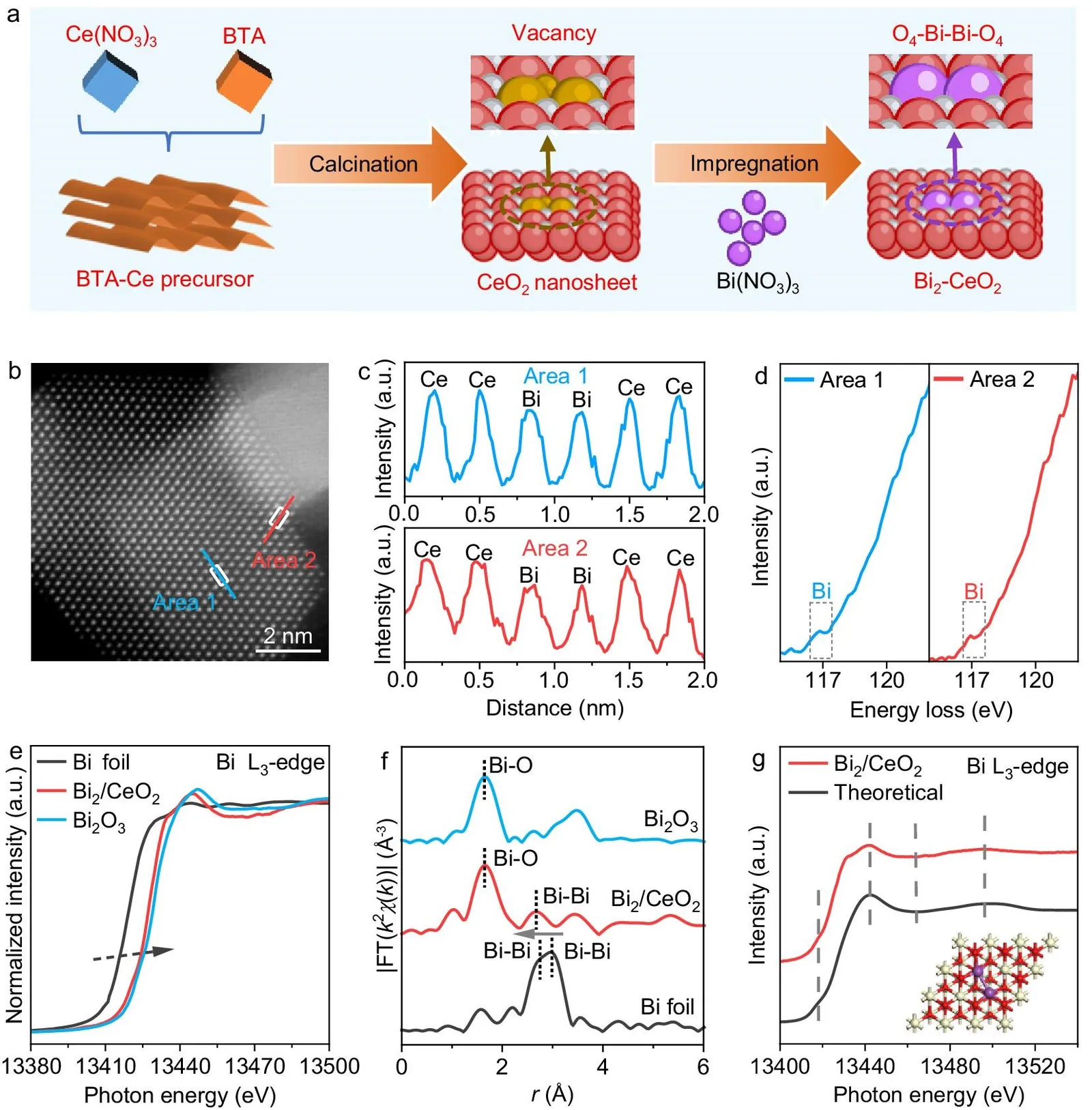
Structural characterization of Bi_2_/CeO_2_. (a) Schematic of the preparation strategy for Bi_2_/CeO_2_. (b) AC-HAADF-STEM image (the Bi atom-pair is highlighted by white regions and marked as Area 1 and Area 2). (c) Brightness intensity of the line profile with Bi atom-pair as a red and blue line highlighted in (b). (d) EELS spectra of Bi atom-pair when the electron beam is placed on Area 1 and Area 2 in the AC-HAADF-STEM image, respectively. (e and f) Normalized XANES spectra and Fourier-transformed EXAFS spectra of Bi_2_/CeO_2_, Bi_2_O_3_ and Bi foil at the Bi L_3_-edge. (g) The experimental (red line) and theoretical (black line) XANES at the Bi L_3_-edge of Bi_2_/CeO_2_.

In this study, the Bi_2_/CeO_2_ catalyst with Bi atoms confined in a vacancy was prepared by a synergistic ultrasonic-assisted chemical impregnation approach (Fig. 3a), employing CeO_2_ nanosheets as the support (Fig. S3, see details in the Supporting Information). Inductively coupled plasma optical emission spectroscopy (ICP-OES) characterization revealed that the actual Bi loading of the as-prepared sample was 1.47 wt% (Table S1). As shown in Fig. S4, the X-ray diffraction (XRD) pattern of Bi_2_/CeO_2_ showed identical diffraction peaks of CeO_2_, with no Bi clusters or Bi oxides being observed. The transmission electron microscopy (TEM) image in Fig. S5a and b revealed a two-dimensional sheet-like morphology of the samples, which exposed the (111) crystal plane. In addition, the energy dispersive X-ray spectroscopy (EDS) elemental mapping images in Fig. S5c demonstrate the uniform distribution of Ce, O and Bi elements in the sample.

To further explore the atomic structure of the Bi_2_/CeO_2_ sample and the distribution of Bi atoms, high-angle annular dark-field scanning transmission electron microscopy (HAADF-STEM) with atomic resolution was employed (Fig. 3b). By exploiting the *Z*-contrast difference between the lighter Bi atoms and the heavier Ce atoms, some isolated dark atom-pairs could be observed (highlighted by white regions). Electron energy loss spectroscopy (EELS) was employed to further demonstrate the elemental composition with greater precision. Figure 3c illustrates the distribution of signal intensity in the specifically highlighted regions, with the Bi–Bi configuration. As seen, EELS analysis of the highlighted regions revealed the characteristic signal of Bi O-edge at 117 eV (Fig. 3d), confirming the presence of the Bi atom-pair. This observation strongly supported the successful formation of constrained Bi atom-pairs through alternative doping on the CeO_2_ surface. Moreover, the Raman peak of the Bi_2_/CeO_2_ in Fig. S6 exhibited a downshift of 2.4 cm^−1^ at ∼460 cm^−1^ (which can be attributed to the Ce–O vibration) and an enhancement of the peak at 609 cm^−1^ (attributed to the Bi–O vibration), indicating the doping of Bi in CeO_2_ [20,21]. These conclusions provided compelling evidence for the successful incorporation of Bi species into the CeO_2_ lattice, conclusively supporting the formation of the proposed ‘O_4_-Bi-Bi-O_4_’ ensemble structure.

X-ray photoelectron spectroscopy (XPS) was employed to investigate the chemical states of elements in Bi_2_/CeO_2_. The high-resolution Bi 4f spectrum exhibited two distinct peaks at 159.1 and 164.3 eV, attributed to Bi*^δ^*^+^ and further confirming the predominance of Bi−O coordination in the local structure (Fig. S7) [22]. For the O 1 s XPS spectrum, three main signal peaks could be classified from high to low binding energy [23]: lattice oxygen (O_lattice_), adsorbed O^−^ or O_2_^2−^ species in O vacancies (O_adsorbed_) and surface-adsorbed OH species. After introduction of Bi atoms, an obvious upshift of 0.3 eV for O_lattice_ peak in the O 1 s XPS spectrum could be observed and the ratio of O_adsorbed_ to all O species decreased from 0.218 to 0.201 (Fig. S8), indicating that the doping Bi atom-pair preferentially occupied the vacancy defects on CeO_2_ nanosheets and induced electronic coupling between Bi atoms and the CeO_2_ support. A similar trend was observable in the Ce 3d XPS spectrum (Fig. S9).

The detailed coordination environment of the Bi atom-pair in Bi_2_/CeO_2_ was investigated by X-ray absorption near-edge structure (XANES) and extended X-ray absorption fine structure (EXAFS). As illustrated in the Bi L_3_-edge XANES spectra, the absorption position of Bi_2_/CeO_2_ lay between those of Bi foil and Bi_2_O_3_, though closer to the latter. This position indicated a high oxidation state of Bi species in Bi_2_/CeO_2_ (Fig. 3e), which is consistent with the Bi*^δ^*^+^ oxidation state observed in the XPS analysis. Moreover, Fourier-transformed EXAFS spectra of Bi_2_/CeO_2_ (Fig. 3f) showed a first coordination shell peak at 2.16 Å, closely matching the Bi–O bond distance in Bi_2_O_3_. Notably, a distinct peak corresponding to the Bi–Bi bond was observed at 3.05 Å, which was shorter than the intra-layer Bi–Bi bond at 3.10 Å in Bi foil [19]. The absence of the characteristic inter-layer Bi–Bi bond peak (3.53 Å) in Bi_2_/CeO_2_ strongly suggested that the observed Bi–Bi scattering path originates from isolated Bi atom-pairs, ruling out the presence of Bi clusters or nanoparticles, which is in excellent agreement with our HAADF-STEM observations. Wavelet transform (WT) analysis of Bi_2_/CeO_2_ revealed distinct intensity at 4.5 and 7.9 Å^−1^, corresponding to the Bi–O bond and Bi–Bi bond, respectively (Fig. S10). These findings were fully consistent with the EXAFS results, providing robust evidence for the unique coordination environment of the Bi atom-pairs in the ‘O_4_-Bi-Bi-O_4_’ ensemble.

To further elucidate the coordination structure of Bi_2_/CeO_2_, quantitative EXAFS fitting analysis was employed. The best-fitted results (Fig. S11 and Table S2) revealed a 4-coordinated Bi–O bond and 1-coordinated Bi–Bi bond, supporting the existence of an ‘O_4_-Bi-Bi-O_4_’ ensemble. To further validate this structural model, the full potential finite difference method (FDM) was employed to theoretically calculate the Bi L_3_-edge XANES spectrum of Bi_2_/CeO_2_ based on the proposed ‘O_4_-Bi-Bi-O_4_’ structure. The calculated spectrum, presented in Fig. 3g, exhibited remarkable agreement with the experimental spectrum, reproducing the main features. To further validate our proposed structure, we systematically evaluated alternative structural configurations, including Bi atom-pairs located at different vacancy sites and in-plane surface positions (Fig. S12). Only the ‘O_4_-Bi-Bi-O_4_’ structure closely matched the experimental Bi L_3_-edge XANES spectrum. Furthermore, the ‘O_4_-Bi-Bi-O_4_’ ensemble exhibited the highest thermodynamic stability among all the ensembles, with a formation energy of −33.55 eV (Fig. S13). Taken together, these comprehensive analyses demonstrated the successful construction of a Bi_2_/CeO_2_ catalyst with the compressed Bi–Bi bond in an ‘O_4_-Bi-Bi-O_4_’ ensemble. For comparative studies, the control samples of Bi_1_/CeO_2_ with isolated Bi single atoms and Bi_*n*_/CeO_2_ containing Bi clusters were also synthesized (Figs S14–S16).

**Figure 4.**
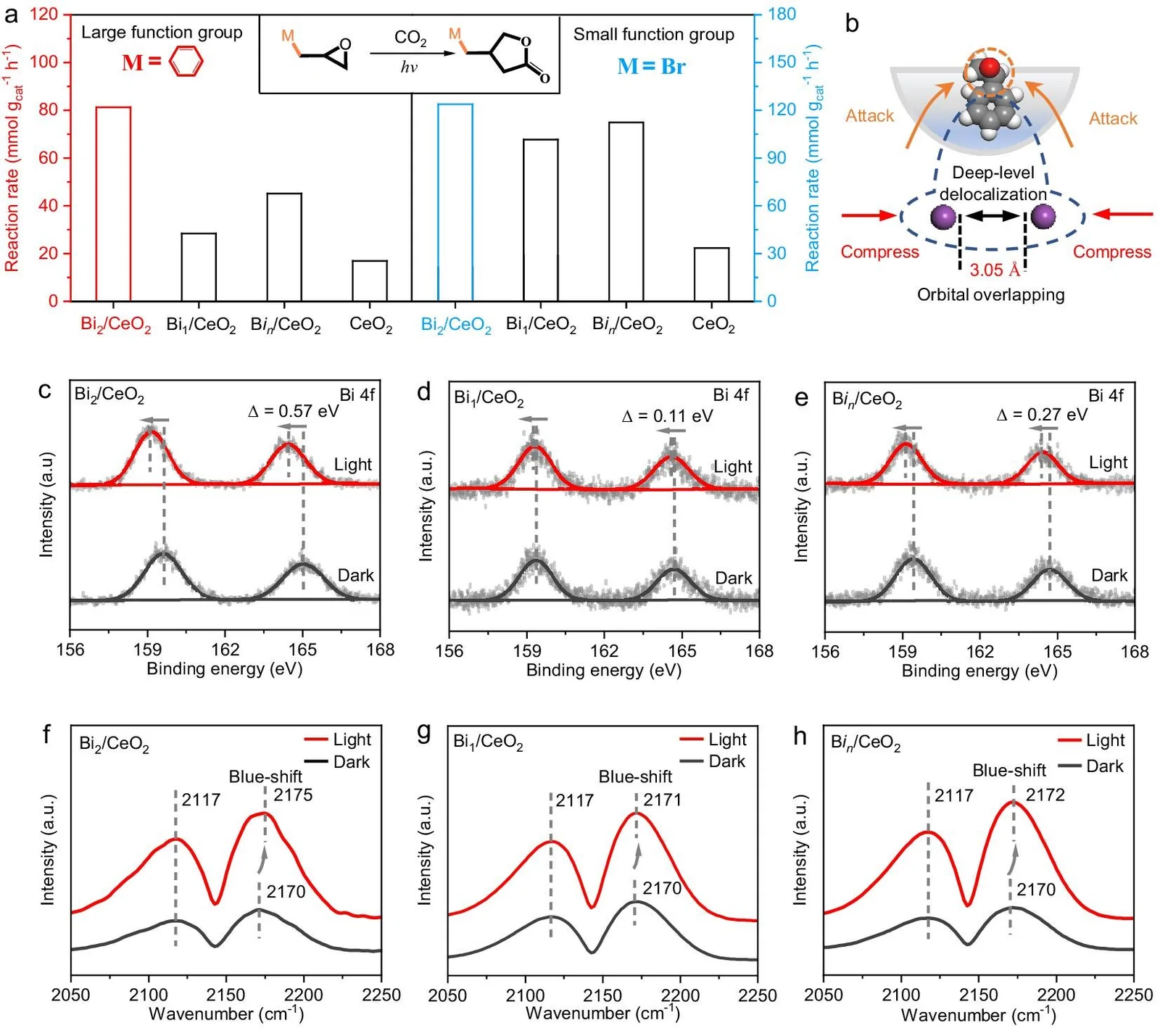
Photocatalytic molecule activation with large/small functional groups, and the investigation of charge-transfer process upon light irradiation for Bi*_x_*/CeO_2_. (a) Photocatalytic performance for styrene oxide and 1-bromo-2,3-epoxypropane cycloaddition. (b) Schematic illustration of Bi_2_/CeO_2_ overcoming steric hindrance in organic molecules with large functional groups. (c–e) *In situ* NAP-XPS spectra of Bi 4f for Bi_2_/CeO_2_, Bi_1_/CeO_2_ and Bi*_n_*/CeO_2_. (f–h) *In situ* CO-DRIFTS for Bi_2_/CeO_2_, Bi_1_/CeO_2_ and Bi*_n_*/CeO_2_ in CO/N_2_ (5%/95%) atmosphere.

Inspired by the aforementioned results, we further investigated the potential of the compressed Bi–Bi bond in the activation of photocatalytic small molecules. The performance of Bi_2_/CeO_2_ for photocatalytic styrene oxide cycloaddition with CO_2_ was tested to explore their ability in overcoming the steric hindrance effect of organics with large functional groups; meanwhile samples of Bi_1_/CeO_2_ and Bi*_n_*/CeO_2_ were tested for comparison (Fig. 4a). Notably, Bi_2_/CeO_2_ exhibited excellent catalytic activity surpassing the majority of other heterogeneous photocatalysts reported thus far (Table S3), with a reaction rate of 81.25 mmol g_cat_^−1^ h^−1^, which was much higher than those of Bi_1_/CeO_2_, Bi_*n*_/CeO_2_ and pristine CeO_2_ nanosheets—28.38, 45.13 and 16.88 mmol g_cat_^−1^ h^−1^, respectively. It is interesting that, when the substrate molecule was changed into small epoxides, such as 1-bromo-2,3-epoxypropane (Br-PO), the reaction rate for all three Bi*_x_*/CeO_2_ catalysts reached a value of >100 mmol g_cat_^−1^ h^−1^, compared with the value of 33.5 mmol g_cat_^−1^ h^−1^ for CeO_2_ nanosheets (Fig. 4a). The discrepancy in catalytic performance under different substrate molecules highlighted the unique capability of Bi_2_/CeO_2_ in overcoming steric hindrance effects (Fig. 4b). Moreover, *in situ* Raman spectroscopy was employed to test the stability of Bi_2_/CeO_2_ under 4-h reaction process with light illumination of 1.2 W/cm^2^ (Fig. S17). Cycling tests were also conducted on Bi_2_/CeO_2_ with no obvious reduction of catalytic performance being observed after five cycles (Fig. S18); meanwhile the structure of the ‘O_4_-Bi-Bi-O_4_’ ensemble remained unchanged (Figs S19–S21).

To further investigate the in-depth photocatalytic mechanism of Bi_2_/CeO_2_ in activating the sterically hindered molecules, *in situ* characterizations in conjunction with DFT calculations were employed. As is known, the photocatalytic styrene oxide cycloaddition began with two simultaneous steps: (i) charge-transfer process upon light irradiation, and (ii) reactant adsorbed on the catalyst, where Bi_2_/CeO_2_ exhibited an exceptional adsorption capacity for styrene oxide as illustrated by theoretical studies. Here, *in situ* near-ambient-pressure XPS (*in situ* NAP-XPS) was then employed to probe the charge-transfer process of the reaction. As depicted by the Bi 4f spectra in Fig. 4c–e, the binding energy of the Bi 4f peak for all three Bi*_x_*/CeO_2_ catalysts downshifted after irradiation, indicating that the electrons transfer from supports (CeO_2_ nanosheets) to Bi sites. Notably, the Bi 4f binding energy of Bi_2_/CeO_2_ exhibited a deeper downshift than that of Bi_1_/CeO_2_ and Bi*_n_*/CeO_2_ (0.57 vs. 0.11 and 0.27 eV). This pronounced shift in Bi_2_/CeO_2_ directly correlates with its deep-level delocalized electronic state, which significantly enhanced charge transfer efficiency after irradiation.

However, no obvious change was observed in the Ce 3d XPS spectra after irradiation (Fig. S22), suggesting no detectable charge-transfer process between Ce atoms and Bi atoms. To gain deeper insights into the subtle charge-transfer dynamics, carbon monoxide (CO) as a sensitive probe molecule was further employed through *in situ* diffuse reflectance infrared Fourier-transform spectroscopy (CO-DRIFTS). This well-established technique allows for precise detection of electron density variations at metal sites with exceptional sensitivity [24,25]. In the CO-DRIFTS spectra of these three Bi*_x_*/CeO_2_ samples (Fig. 4f–h), the high- and low-frequency bands were ascribed to the CO linear adsorption on the interface Ce atom (Ce-O-Bi) and the lattice Ce atom (Ce-O-Ce), respectively [26,27]. After irradiation, a blue shift was observed in the high-frequency band, while the low-frequency band remained unchanged. This phenomenon indicated electron transfer from interfacial Ce atoms to Bi atoms, resulting in reduced electron density at the interface Ce sites. Consequently, the weakened back-donation to the 2π* antibonding orbital of adsorbed CO leads to the observed blue shift in the ν(CO) frequency. Importantly, Bi_2_/CeO_2_ exhibited a more pronounced blue shift compared to Bi_1_/CeO_2_ and Bi*_n_*/CeO_2_, further corroborating its superior charge transfer capability under illumination. Based on *in situ* characterizations, it is safe to conclude that Bi_2_/CeO_2_ exhibited an enhanced charge-transfer process with styrene oxide under irradiation. Benefiting from the formation of deep-level delocalized Bi-*p* orbitals, Bi_2_/CeO_2_ was able to offer photogenerated electrons with sufficient electron energy to overcome the steric hindrance effect from large functional groups and activate the substrate molecules.

**Figure 5.**
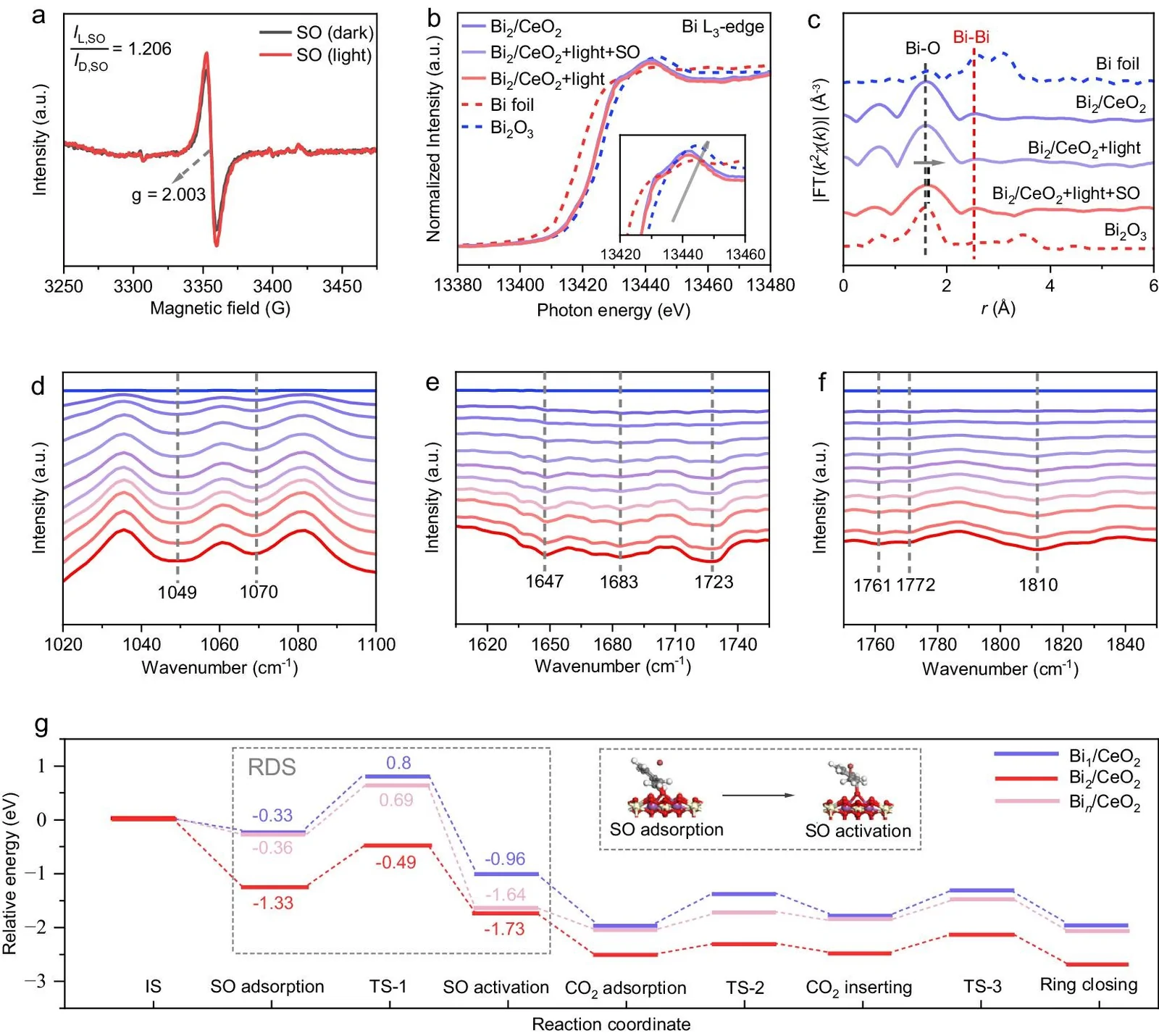
Mechanistic investigations of Bi_2_/CeO_2_ in photocatalytic styrene oxide activation and cycloaddition. (a) *In situ* low-temperature ESR spectra of Bi_2_/CeO_2_ before and after the introduction of styrene oxide. (b and c) *In situ* Bi L_3_-edge XANES and EXAFS spectra of Bi_2_/CeO_2_ under different reaction conditions. (d–f) *In situ* DRIFTS of Bi_2_/CeO_2_ for the photocatalytic styrene oxide cycloaddition. (g) Potential energy profiles of styrene oxide cycloaddition along the reaction pathway for Bi_1_/CeO_2_, Bi_2_/CeO_2_ and Bi*_n_*/CeO_2_, respectively. (Bi_8_/CeO_2_ was selected for the model of Bi*_n_*/CeO_2_).

To go further, *in situ* electron spin resonance (ESR) spectra were employed to examine the substrate-molecule activation process in Bi_2_/CeO_2_. The characteristic symmetric signal of Lorentzian lines with *g* values of 2.003 corresponds to the unpaired electrons in the tested systems, where the intensity of the signal under light (*I*_L_) and dark (*I*_D_) conditions were marked [26,27]. As shown in Fig. 5a, the introduction of styrene oxide under inert atmosphere resulted in a significant increase in the *I*_L,SO_/*I*_D,SO_ ratio (1.206). This enhancement could be ascribed to the activation of styrene oxide at the active site, where the opening of the three-membered ring structure generated reaction intermediates with increasing unpaired electron density. In comparison, the ESR signal for the presence of N_2_ or CO_2_ showed little change (Fig. S23), confirming the specific nature of styrene oxide activation. *In situ* XANES spectra of the Bi L_3_-edge were employed to obtain the subtle change in oxidation states of Bi atoms under reaction conditions. As illustrated in Fig. 5b, the intensity of the white line for the Bi L_3_-edge exhibited a decline following illumination, indicating a reduction in oxidation states. This finding was consistent with the conclusion drawn from the above *in situ* NAP-XPS (Fig. 4c), suggesting the accumulation of photoelectrons on Bi atoms under light. However, in the presence of styrene oxide, the white line intensity increased, indicating electron transfer from Bi atoms to the activated substrate. This redox behavior provided direct evidence for the catalytic role of Bi atoms in substrate activation. The local coordination environments of Bi atoms under reaction conditions are shown in Fig. 5c. After the introduction of styrene oxide, the adsorption and activation process induced a 0.05 Å elongation of the primary Bi–O bond, while the compressed Bi–Bi bond remained unchanged at 3.05 Å throughout the reaction. This structural stability of the Bi–Bi bond under reaction conditions underscored the robustness of the ‘O_4_-Bi-Bi-O_4_’ ensemble in Bi_2_/CeO_2_, while the dynamic changes in Bi–O bonding demonstrated the effective participation of Bi sites in substrate activation.

As a powerful technique for elucidating the reaction mechanism, *in situ* CO-DRIFT was then conducted to determine the intermediates under reaction conditions. As demonstrated in Fig. S24, a series of characteristic peaks emerged from 1000 cm^−1^ to 2000 cm^−1^. The peaks at 1049 cm^−1^ and 1070 cm^−1^ could be attributed to the symmetric stretching vibrations of C–O species (Fig. 5d) [30], while the peak at 1450 cm^−1^ could be ascribed to the twisting vibrations of –CH_2_ (Fig. S22) [31], which suggested the opening of the three-membered ring of styrene oxide on Bi_2_/CeO_2_ and the formation of carbonate intermediate. The peaks at 1647 cm^−1^, 1683 cm^−1^ and 1723 cm^−1^ demonstrated the presence of C=O vibrations, indicating CO_2_ insertion into the carbonate intermediate (Fig. 5e) [32]. The symmetric split stretching vibration of carbonyl [33] in products could be observed at 1761 cm^−1^ and 1810 cm^−1^ (Fig. 5f). The potential reaction mechanism of the photocatalytic styrene oxide cycloaddition on Bi_2_/CeO_2_ is depicted in Fig. S25, which presents a comprehensive overview of the process. To complement our experimental findings, DFT calculations were further employed to compare the reaction pathways on Bi_1_/CeO_2_, Bi_2_/CeO_2_ and Bi*_n_*/CeO_2_ (Fig. 5g). The RDS for styrene oxide cycloaddition on Bi*_x_*/CeO_2_ was identified as the activation step of styrene oxide, where the Bi_2_/CeO_2_ exhibited a lower energy barrier than Bi_1_/CeO_2_ and Bi*_n_*/CeO_2_. All *in situ* experiments and DFT calculations conclusively demonstrated that the compressed Bi–Bi bond in Bi_2_/CeO_2_ induced deep-level delocalization of Bi electron states. This unique electronic structure facilitated the efficient charge-transfer process under irradiation, enabling the catalyst to effectively overcome the steric hindrance effects of the phenyl group and activate the substrate molecules.

## CONCLUSION

In summary, we propose here a vacancy-constrained strategy achieved by compressing bonds of supported metal atoms in a confined surface vacancy to design a catalyst with a deep-level delocalization character, thus overcoming the steric hindrance effect of molecules. By taking Bi_2_/CeO_2_ as an example, we show that the compressed Bi–Bi bonds exhibit strong penetrating capability that overcomes the steric hindrance effect of large functional groups by enhancing the charge-transfer process between the active sites and substrates. Systematic *in situ* characterizations with DFT calculations revealed that irradiation induces the accumulation of deep-level delocalized photogenerated electrons on Bi atom-pair. These energetic electrons effectively penetrate the *π*-bond electron clouds of the phenyl group, subsequently activating the three-membered ring of styrene oxide. This activation process was identified as the rate-determining step (RDS) in the styrene oxide cycloaddition reaction. As a result, the Bi_2_/CeO_2_ catalyst demonstrates enhanced photocatalytic styrene oxide cycloaddition reactions without any solvents, which is three times and two times higher in catalytic activity than that of a Bi single-atom catalyst and Bi cluster catalysts, respectively. This work establishes a new paradigm for designing efficient heterogeneous photocatalysts capable of overcoming steric hindrance limitations in the synthesis of high-value-added chemicals.

## Supplementary Material

nwag388_Supplemental_File
