# Supplementary material for: Breaking steric hindrance restriction: compressed metal bonds realizing deep-level orbital delocalization
Source: Natl Sci Rev. 2026 Jul 6;13(18):nwag388. doi: 10.1093/nsr/nwag388 (PMC13613982; doi:10.1093/nsr/nwag388)
Supplement: Supplementary file 1 [file nwag388_supplemental_file.pdf]

Supporting Information

Breaking steric hindrance restriction: compressed metal bonds realizing deep-level orbital delocalization

Hanghao Ying[1,†], Liuxin Xu[1,†], Xia Zhong[1], Yi Liu[1], Xiaodong Zhang[1,*], Yi Xie[1,2,*]

[1]Hefei National Research Center for Physical Sciences at the Microscale, University of Science and Technology of China, Hefei 230026, China and [2]State Key Laboratory of Precision and Intelligent Chemistry, University of Science and Technology of China, Hefei 230026, China

***Corresponding authors.** E-mails: zhxid@ustc.edu.cn; yxie@ustc.edu.cn

[†]Equally contributed to this work.

Experimental section:

Synthesis of CeO_2 nanosheet

The CeO_2 nanosheet was synthesized by a modified hydrothermal reactions.[1] In detail, 2.17 g $Ce(NO_3)_3 \cdot 6H_2O$ and 1.27 g 1,2,4,5-Benzenetetracarboxylic acid (BTA) were dissolved in 75 mL deionized water and 75 mL ethanol, respectively. Both solutions were stirred in a three-necked flask with the temperature at 80 °C for 60 min. After slowly cooling to room temperature, precipitate of BTA-Ce precursor was collected by centrifugation with the washing by ethanol and deionized water for several times and then dried at 75 °C for 8 hours. The CeO_2 nanosheet was obtained by the calcination of the BTA-Ce precursor at 600 °C in muffle furnace for 3h.

Synthesis of Bi_2/CeO_2 nanosheet

Bi_2/CeO_2 nanosheet was synthesized by an ultrasonic and chemical impregnation method. In detail, 500 mg CeO_2 nanosheet was dispersed into 50 mL deionized water with the vigorous ultrasonic treatment for 9 h. Then 23.2 mg $Bi(NO_3)_3 \cdot 5H_2O$ was added into the mixture and stirred for 4 h. The product was collected by centrifugation and washed by deionized water and ethanol for 3 times, then placed it in a vacuum oven at 60 °C for 12 h. Finally, the products were heated at 500 °C for 3 h in air to obtain the Bi_2/CeO_2 nanosheet.

Synthesis of Bi_1/CeO_2 and Bi_n/CeO_2 nanosheet

Bi_1/CeO_2 and Bi_n/CeO_2 nanosheet was synthesized by traditional chemical impregnation. Typically, 500 mg CeO_2 nanosheet was dispersed into 50 mL deionized water with vigorous stirring and then 5.8 mg and 92.8 mg $Bi(NO_3)_3 \cdot 5H_2O$ were respectively added into the mixture. After 4 h stirring, the product was collected by centrifugation and accompanied with washing by deionized water and ethanol for several times, and then placed it in a vacuum oven at 60 °C overnight. Bi_1/CeO_2 was then heated at 500 °C and Bi_n/CeO_2 was heated at 700 °C respectively for 3 h in air to obtain the Bi_1/CeO_2 and Bi_n/CeO_2 nanosheets.

Catalyst characterization

The Bi loading content was determined by inductively coupled plasma atomic emission spectrometry (ICP-AES) on Atomscan Advantage instrument (Thermo Jarrell Ash Corporation). HAADF-STEM images and corresponding energy dispersive spectroscopy (EDS) mapping were performed on a JEOL JEM-ARM200F TEM/STEM. Electron energy loss spectroscopy (EELS) was

performed on a Gatan Quantum 1066. Powder X-ray diffraction patterns (PXRD) were carried out on a Japan Rigaku MiniFlex 600 equipped with graphite-monochromated Cu Kα radiation (λ = 1.54178 Å). The transmission electron microscopy (TEM) was observed with a JEOL JEM-ARM200F field emission electron microscope at an acceleration voltage of 200 kV. High resolution transmission electron microscopy (HRTEM) was carried out on a JEOL JEM-ARF200F. Scanning electron microscopy (SEM) images were taken on a FEI Sirion-200 field emission scanning electron microscope operated at 5 kV. X-ray photoelectron spectroscopy (XPS) valence spectra were acquired on an ESCALAB MKII spectrometer with Mg Kα excitation source (hv = 1253.6 eV). The binding energies derived from the XPS analysis were corrected against the specimen charging by referencing C 1s to 284.8 eV. Room-temperature ESR spectra were collected using a JEOL JES-FA200 electron spin resonance spectrometer (298 K, 9.062 GHz). The electrochemical measurements were performed on an electrochemical workstation (CHI760E, Shanghai Chenhua Limited, China).

Catalytic test

In general, a mixture of 20 mg catalyst, 10 mmol styrene oxide, 0.25 mmol tetrabutyl ammonium bromide (TBAB), was placed into a 20 mL quartz bottle and an injector filled with CO_2 was connected to the photo-reactor. The reactor was purged with CO_2 for 2 min to replace the air in the quartz bottle for the reaction. The catalytic cycloaddition reaction was performed under light irradiation with Xe lamp (CEL-HXF300-T3, Beijing China Education Au-light Technology Co., Ltd.). After the reaction, the liquid products were detected by NMR (400-MHz Bruker AVANCE AV III) spectroscopy with addition of internal standard (25 μl 1,1,2,2-Tetrachloroethane).

XAFS measurements

The X-ray absorption data at the Bi L_3-edge of the samples were recorded at room temperature in transmission mode using ion chamber at the BL20U1 Hard branch of energy material beamline (E-line) of Shanghai Synchrotron Radiation Facility in the 3.5 GeV storage ring, with injection currents of 220 mA. In the measurement, a Si (111) double-crystal monochromator was used. Bi foil was used as reference samples and measured in the transmission mode. For in situ XAFS spectra of Bi_2/CeO_2 under reaction conditions, the sample was first pressed into plate and loaded into a specially designed hexagonal reactor with polyimide windows. The measure was measuring in a

room temperature with a 300 W xenon lamp. The XAFS data were processed using the ATHENA module implemented in the IFEFFIT software packages[2], and the least-square curve-fitting was accomplished by using the ARTEMIS module of IFEFFIT[3].

***In situ* DRIFTS measurements**

In situ diffuse reflectance infrared Fourier-transform spectroscopy (DRIFTS) measurements were performed by employing a Bruker IFS 66v Fourier-transform spectrometer equipped with a Harrick diffuse reflectance accessory at the Infrared Spectroscopy and Microspectroscopy Endstation (BL01B) in NSRL in Hefei, China. The samples were held in a custom-fabricated IR reaction chamber which was specifically designed to examine highly scattering powder samples in the diffuse reflection mode. The chamber was sealed with two ZnSe windows.

***In situ* ESR measurements**

In situ ESR spectra was obtained using a JEOL JES-FA200 spectrometer (133 K, 9.098 GHz). Microwave power employed was 1 mW; sweep width ranged from 299.5 to 349.5 mT. Modulation frequency and modulation width were 100 kHz and 0.35 mT. Specifically, the in situ ESR measurement was performed at 133 K in different atmospheres with light irradiation (Xe lamp with full spectrum).

***In situ* NAP-XPS measurements**

For *in situ* NAP-XPS. The catalyst was dropped onto a clean silicon pellet, and dried at room temperature. After the sample was transferred to the analysis chamber, the spectra were firstly collected in UHV. Subsequently, the surface of the catalyst was exposed to light from a xenon lamp for 60 minutes, after which the spectra were recorded.

***In situ* Raman measurements**

In situ Raman spectra were taken on a WITec alpha 300R confocal Raman microscope with a 600 grooves/mm diffraction grating. A 532 nm excitation laser was used with an exact power of 4 mW. Calibration was performed based on the peak at 520 cm^{-1} of a silicon wafer standard. The sample was first pressed into sheet and loaded into a specially designed reactor with sapphire windows. A 532 nm laser vertically irradiates the sample at a 90 ° angle and reaction light from the xenon lamp inject from the side at a 45° angle.

Computational methods

Density functional theory (DFT) calculation was performed using the Vienna ab initio Simulation Package[4]. The Perdew–Burke–Ernzerhof (PBE)[5] exchange-correlation functional within the generalized gradient approximation (GGA) was employed to describe the exchange-correlation energy. The projector-augmented-wave (PAW)[6] method was adopted for the pseudopotentials. The energy cutoff for the plane wave basis expansion was set to 450 eV. The force and energy change for all atomic structures were set as 0.02 eV/Å and 10^{-5} eV for the convergence criterion, respectively. The Bi_x/CeO_2 models used for the simulation were all constructed in four atomic-layered 3×3 supercell, with vacuum layer of 15 Å in the z direction to avoid the periodic interaction. During the further calculation, the bottom two layers of CeO_2 were fixed to simulate the bulk condition. It is noteworthy that, the Bi atom-pair in Bi_2/CeO_2 model was confined at the Ce-O-Ce trimer-vacancy for the stability of the structure. The Bi atom-pair located on the surface of CeO_2 (111) was found to be unstable after structural optimization and transformed into two separate Bi single atoms. Besides, the Bi species in other Bi_x/CeO_2 models were located on surface of CeO_2 (111) with good stability. A Hubbard U parameter of 4.5 eV was added to the cerium 4f orbitals within the Dudarev formulation[7]. Different Hubbard parameters lead to differentiated properties in matching experiment and calculation results, where values of 2-3 eV are needed for getting correct thermochemistry[8-9] while 4.5-5 eV is well suited to correct electron localization of electrons in the 4f state in reduced CeO_2[10-12]. The theoretical Bi L_3-edge X-ray absorption near edge structure (XANES) spectra for the Bi_2/CeO_2 models were generated using self-consistent, real-space full-multiple-scattering and the full-potential finite difference method together with the FDMNES code[13-15]. Transition state searching was determined using the climbing-image nudged elastic band (CI-NEB) method[16].

Supplementary Figures

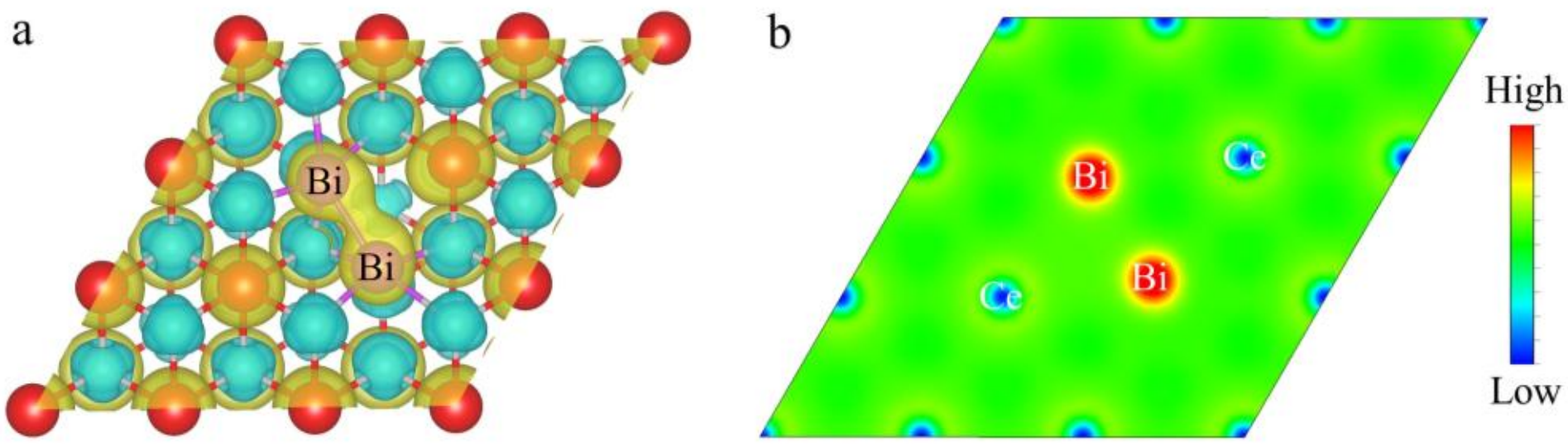

Figure S1. (a) The difference density of electron distribution for Bi_2/CeO_2 and (b) The corresponding electron-density isosurface on (001) direction.

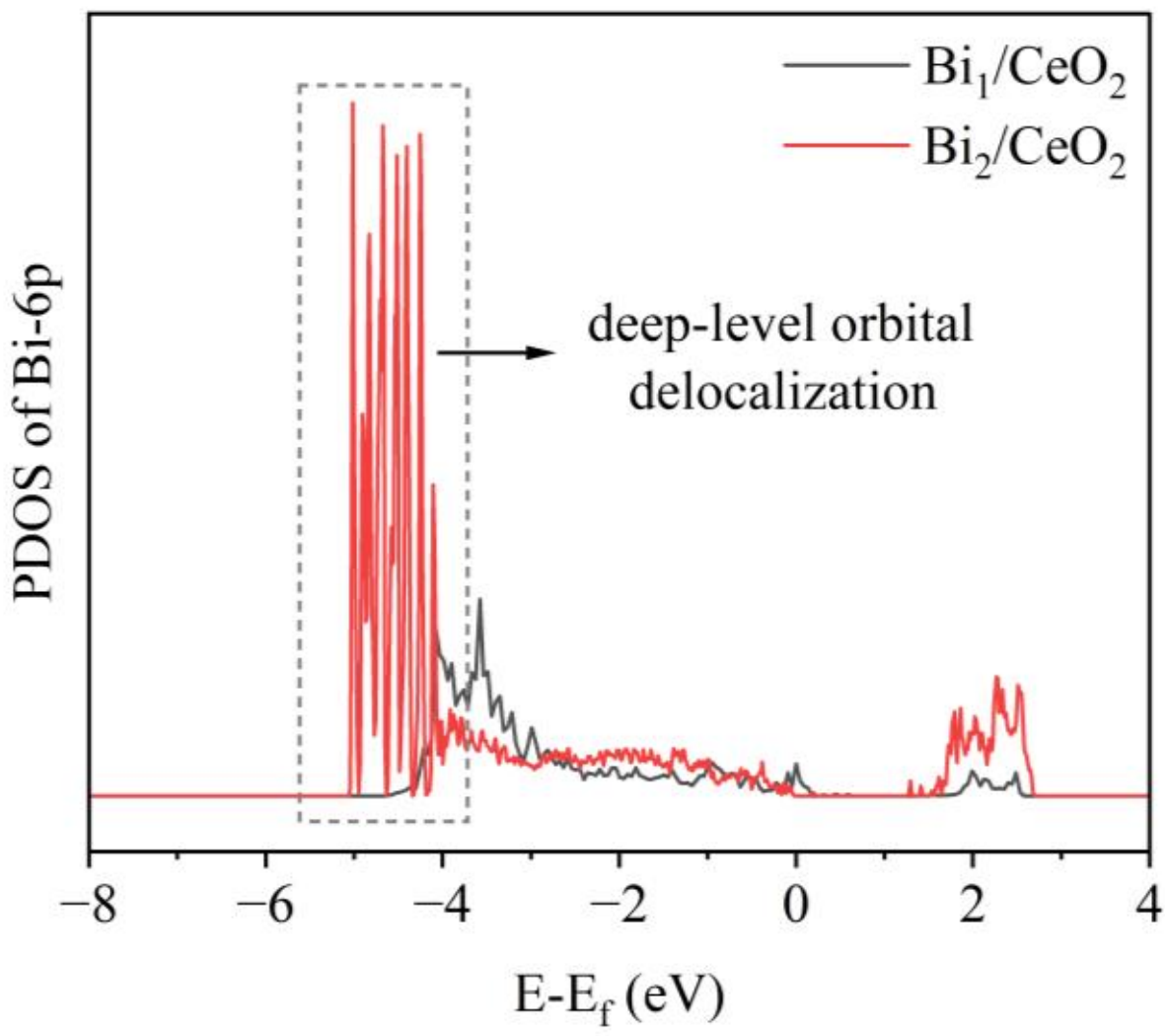

Figure S2. The PDOS analysis of Bi_2/CeO_2 and Bi_1/CeO_2.

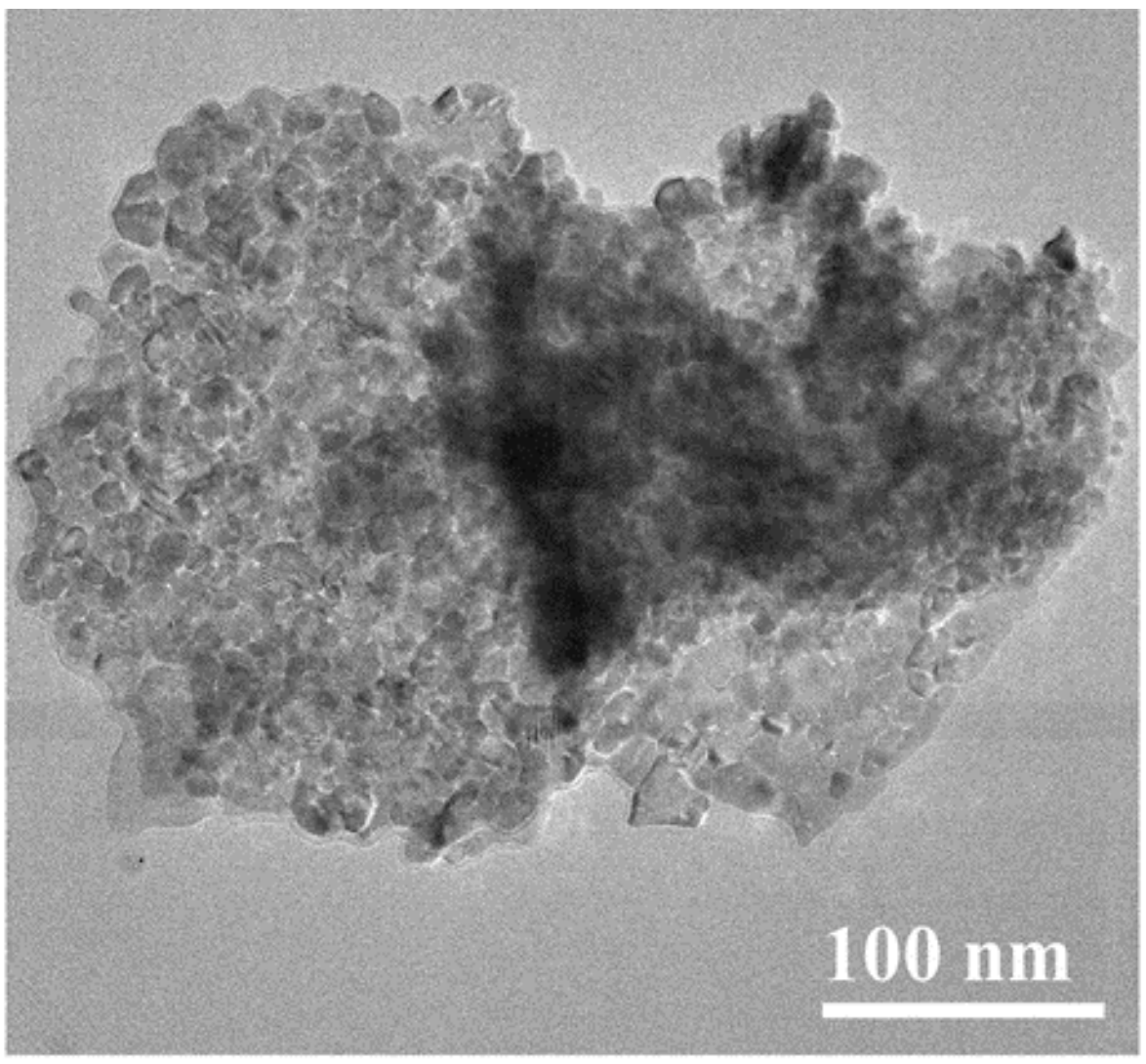

Figure S3. TEM images of CeO_2 nanosheet.

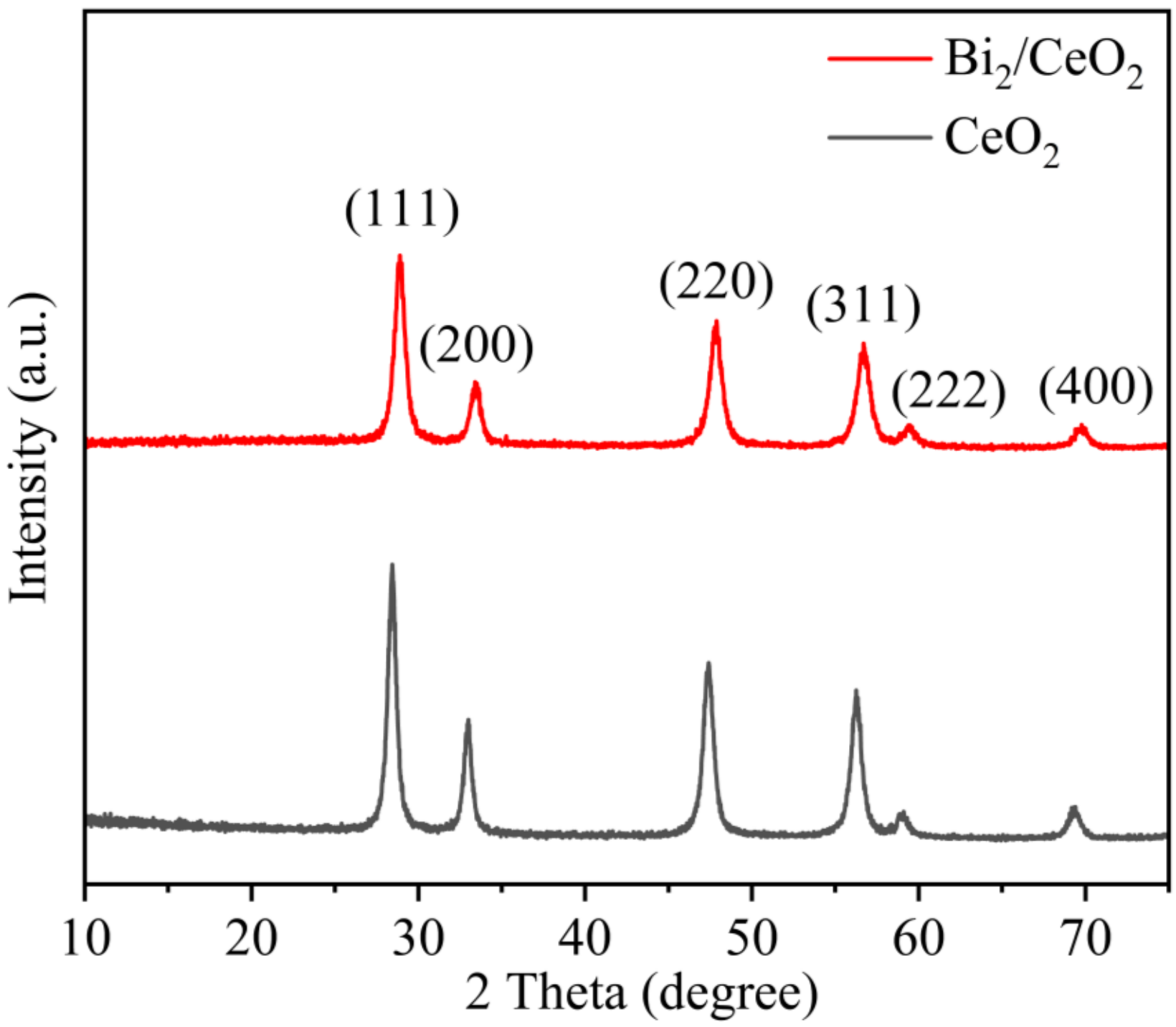

Figure S4. XRD pattern of CeO_2 nanosheet, and Bi_2/CeO_2.

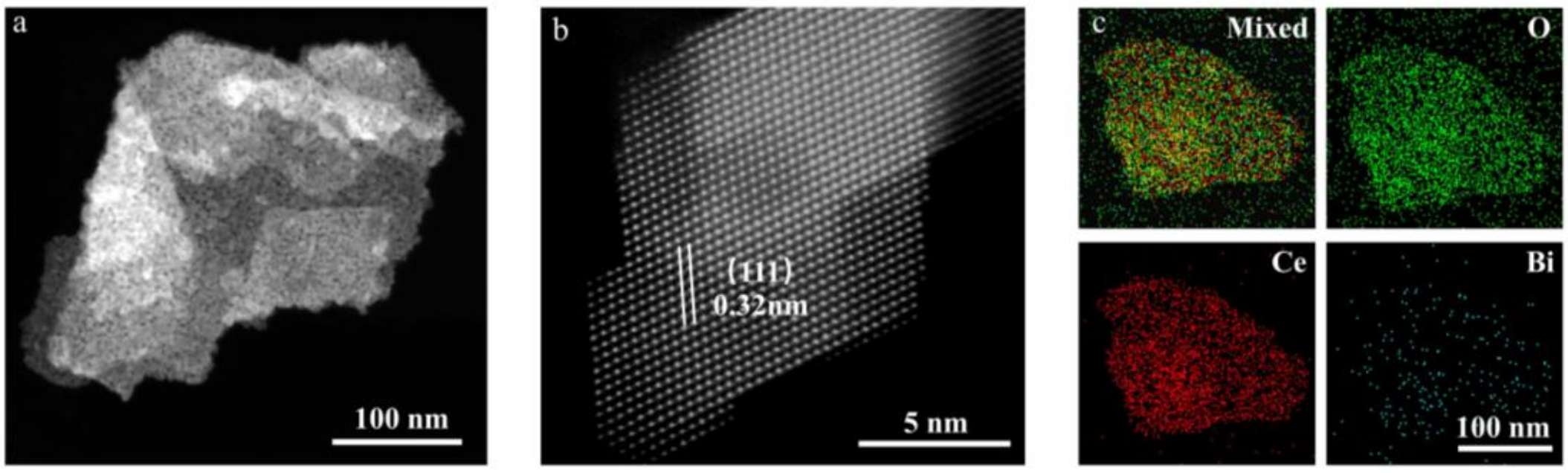

Figure S5. (a) TEM images of Bi_2/CeO_2. (b) HRTEM images of Bi_2/CeO_2. (c) STEM elemental mapping of Bi_2/CeO_2 (green, O; red, Ce; blue, Bi).

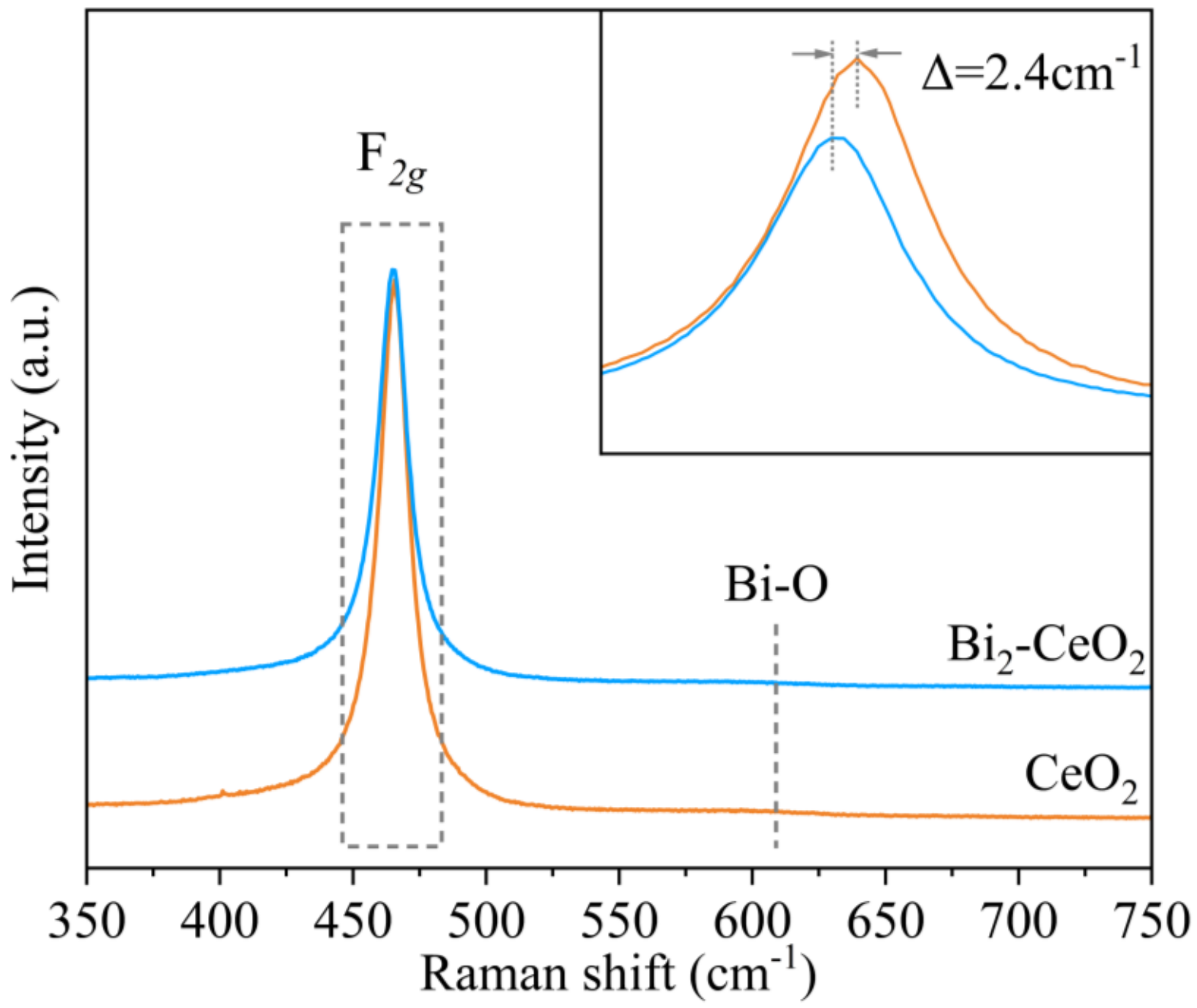

Figure S6. Raman spectra of CeO_2 nanosheet, and Bi_2/CeO_2.

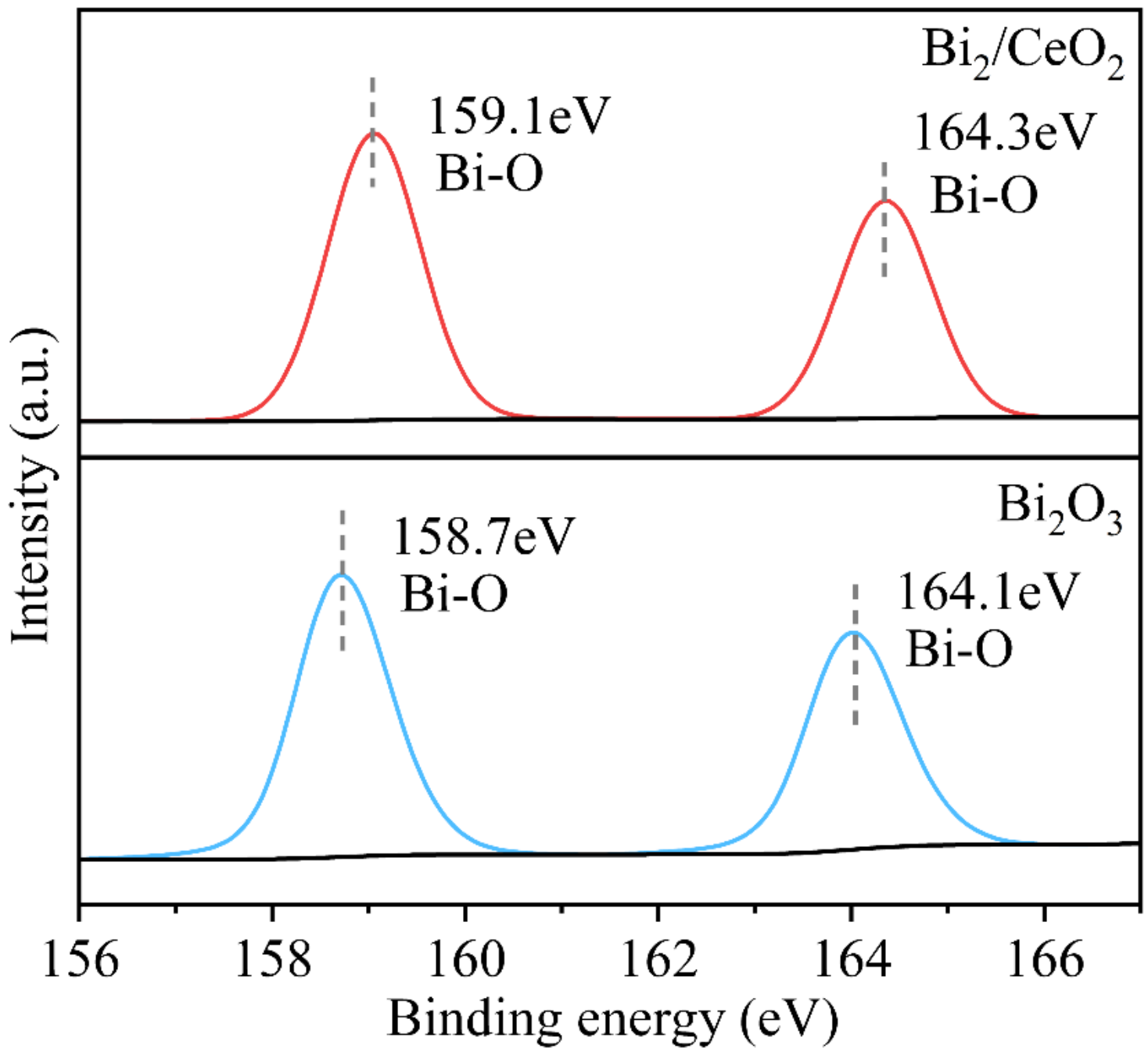

Figure S7. Bi 4f XPS spectra for Bi_2/CeO_2 and Bi_2O_3.

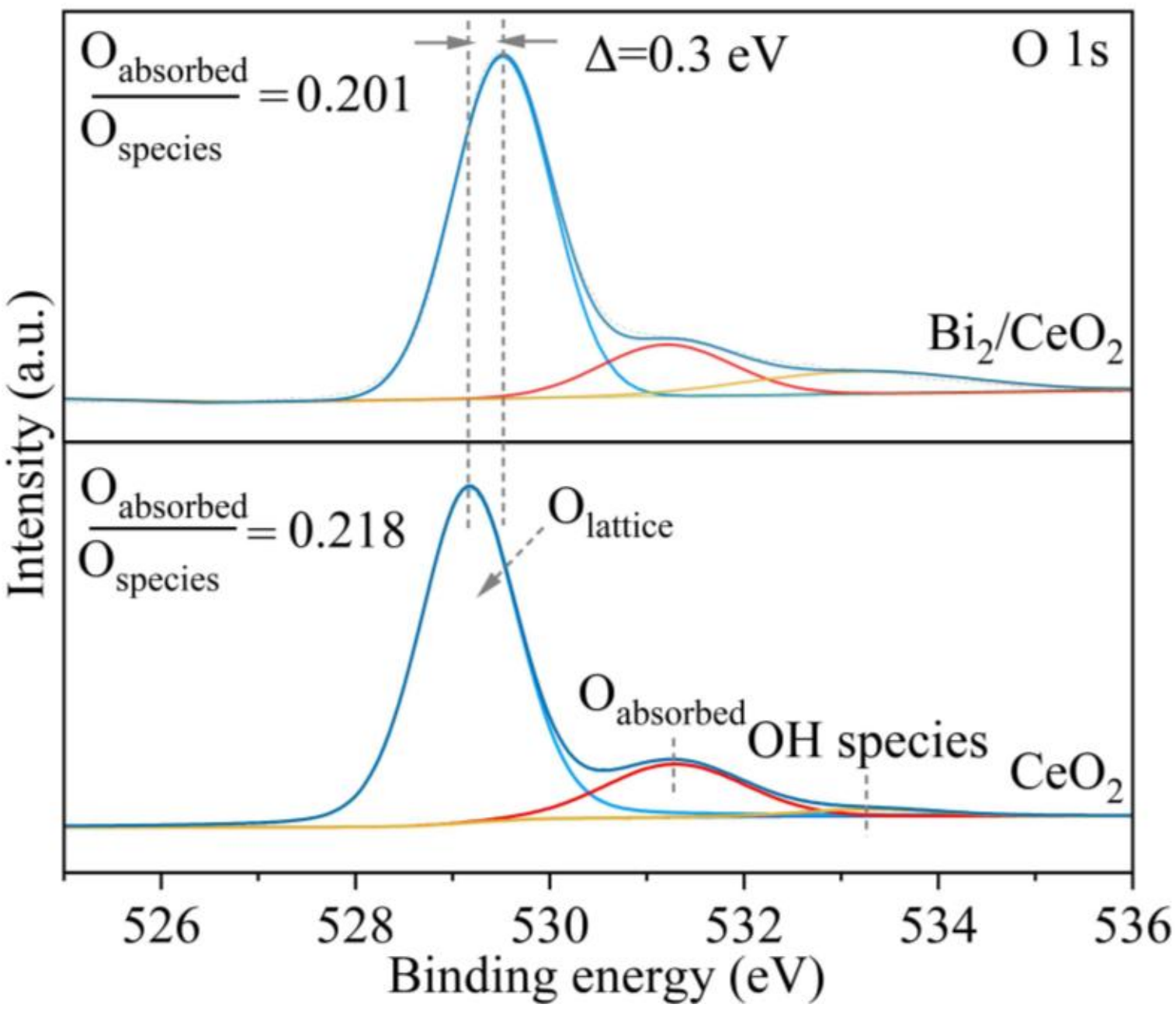

Figure S8. O 1s XPS spectra for Bi_2/CeO_2 and CeO_2 nanosheet.

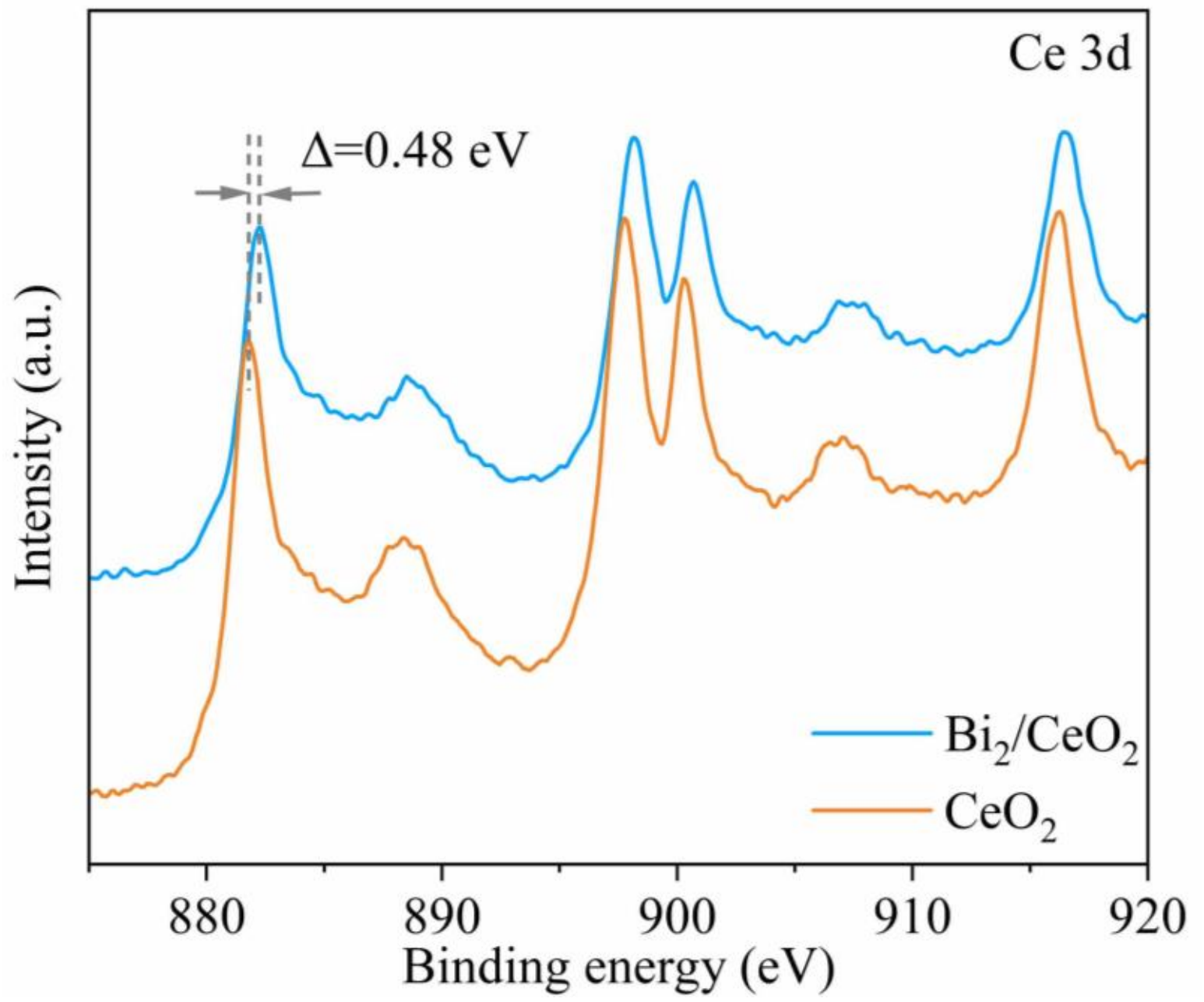

Figure S9. Ce 3d XPS spectra for Bi_2/CeO_2 and CeO_2 nanosheet.

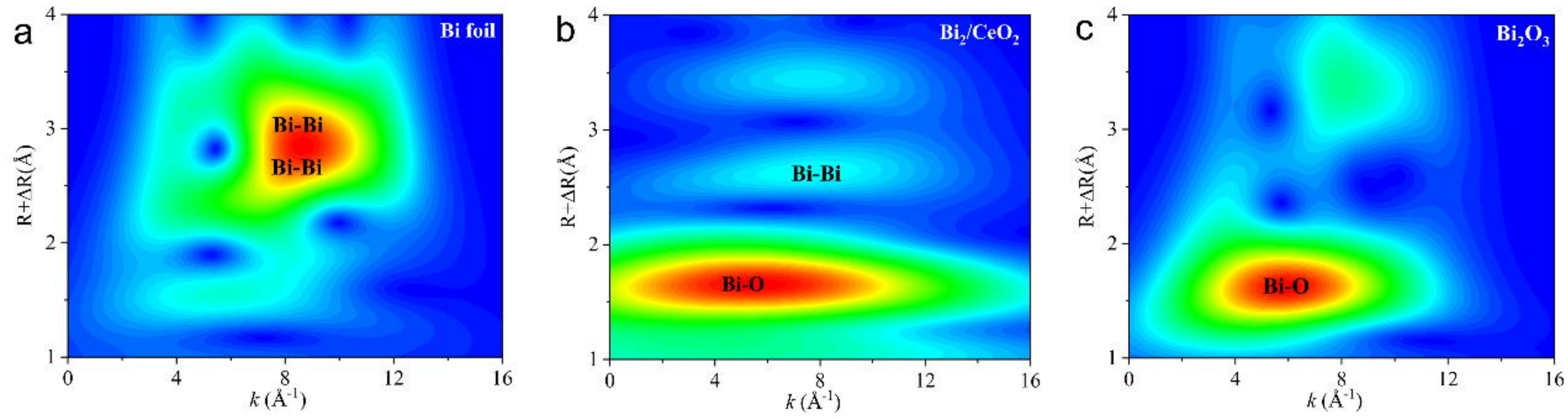

Figure S10. Wavelet transform simulation for the k_3-weighted EXAFS signals for Bi foil, Bi_2/CeO_2, and Bi_2O_3, respectively.

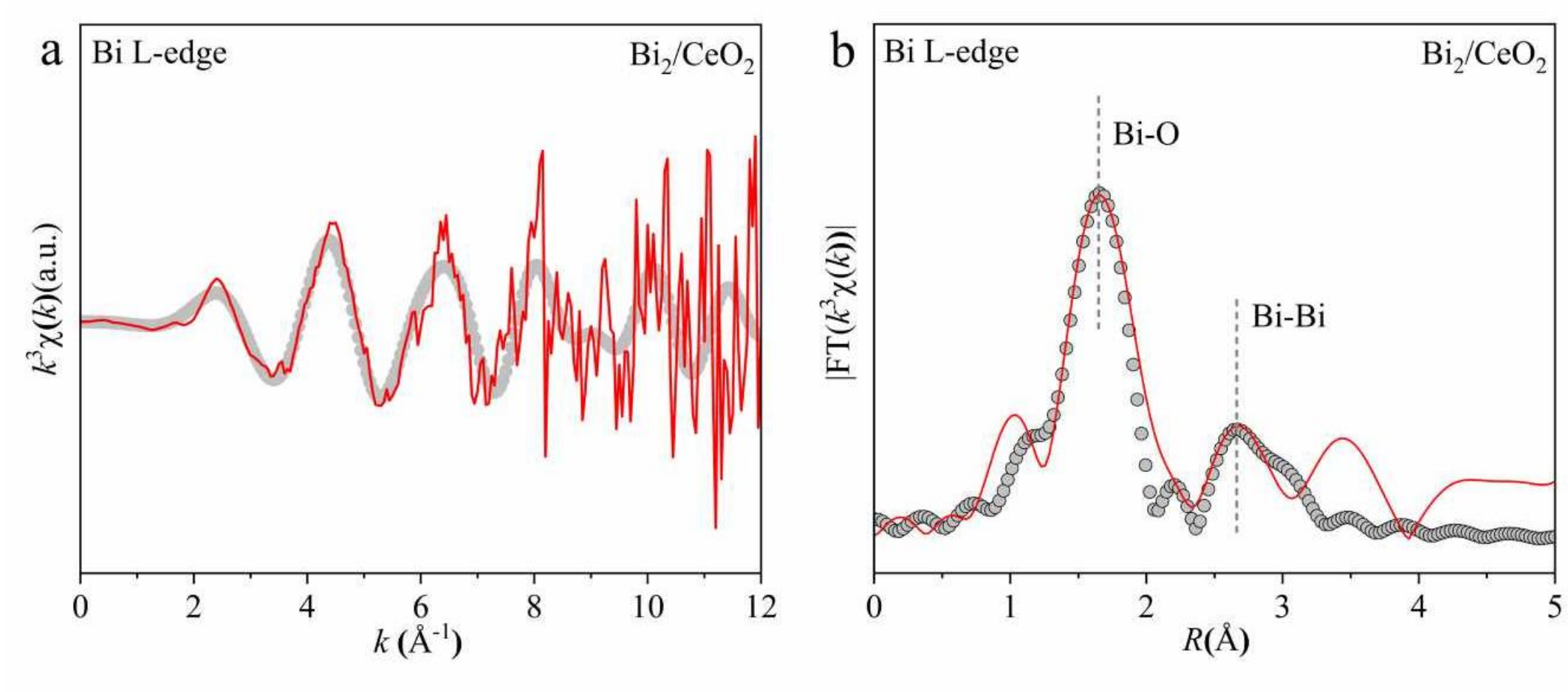

Figure S11. (a) The fitting of the EXAFS χ(k) for Bi_2/CeO_2. (b) The fitting of the Fourier-transforms of the k3-weighted EXAFS for Bi_2/CeO_2.

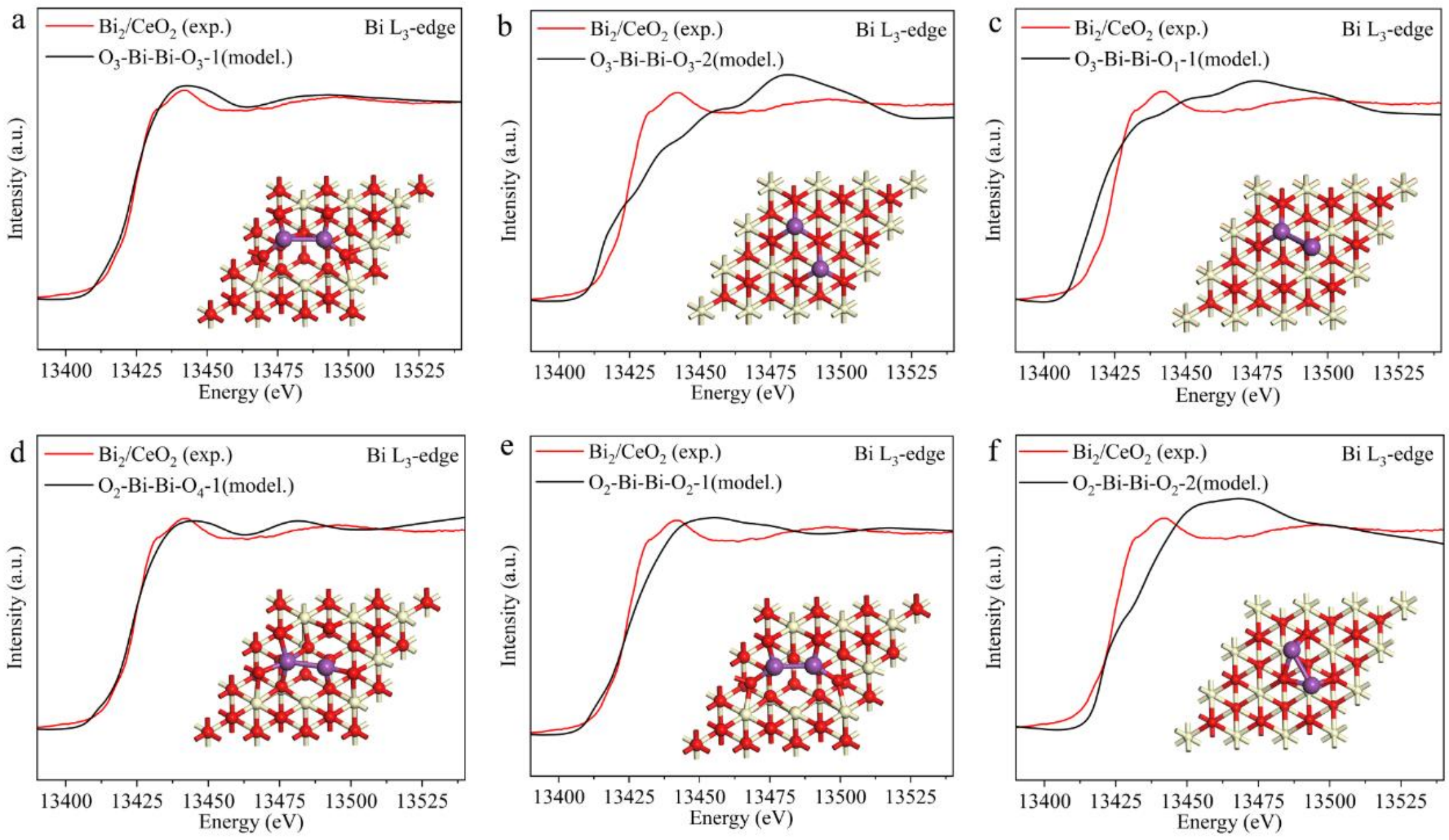

Figure S12. Comparison of experimental Bi L_3-edge XANES spectrum (red line) with theoretically calculated spectra (black line) of various possible models. The theoretical XANES spectra were calculated using the full potential finite difference method. Herein, the experimental Bi L_3-edge XANES spectrum were use the same value in comparison.

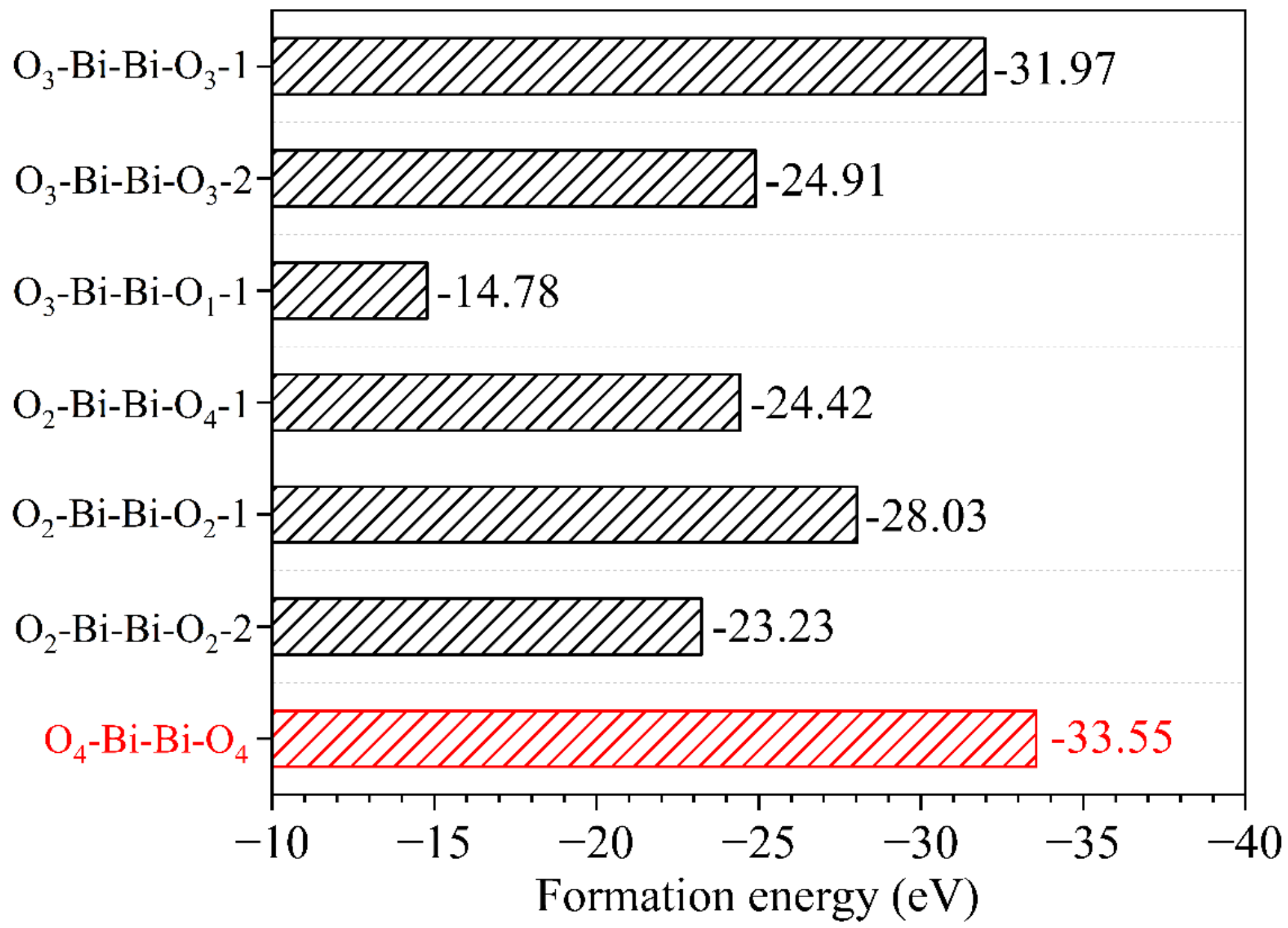

Figure S13. The formation energy of seven models in Figure 2f and Figure S12 for Bi_2/CeO_2. The structure of "O_4-Bi-Bi-O_4" exhibits the lowest formation energy with the highest thermodynamic stability.

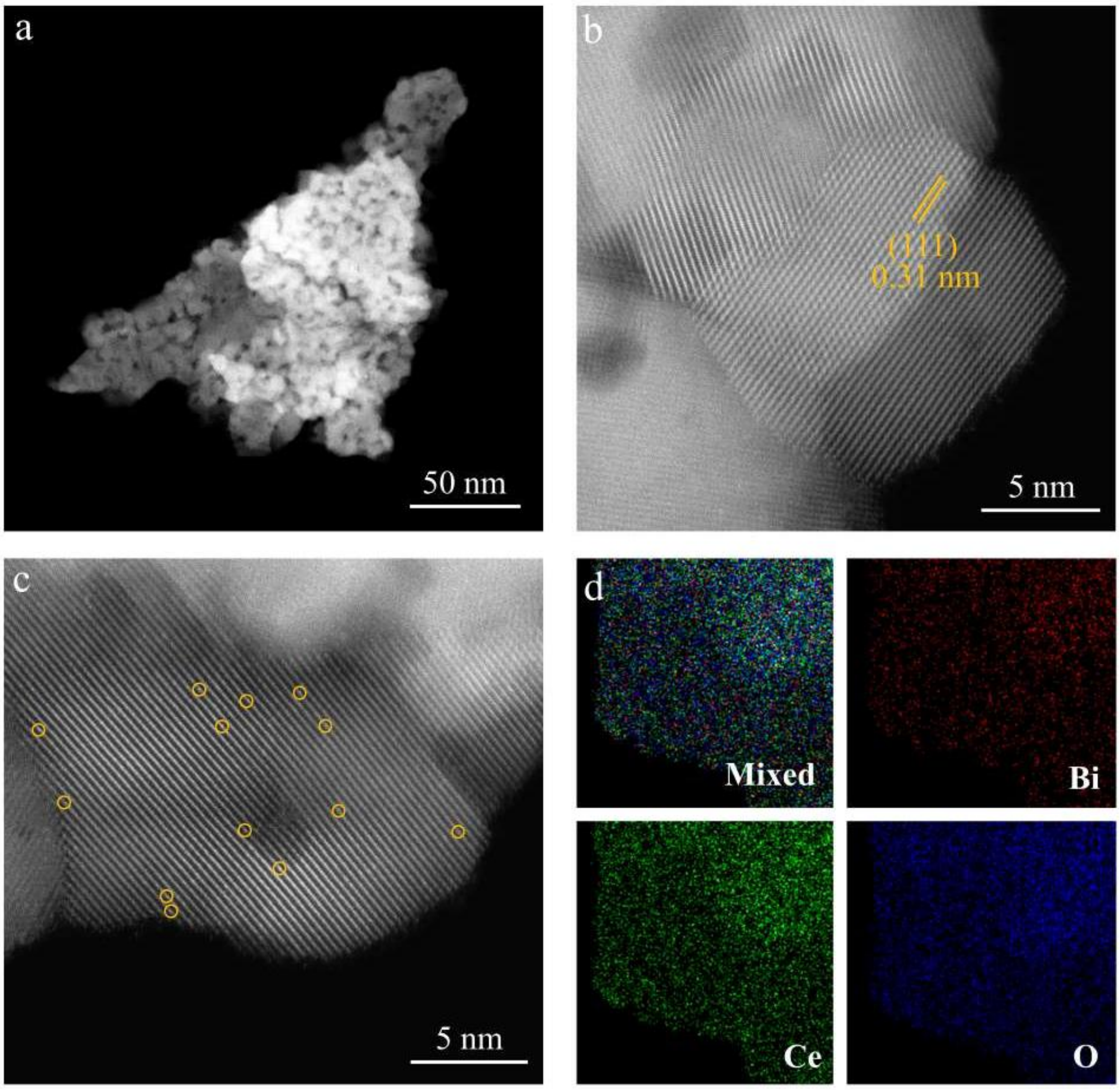

Figure S14. (a) TEM images of Bi_1/CeO_2. (b) HAADF-STEM images of Bi_1/CeO_2 (e) AC-HAADF-STEM image of Bi_1/CeO_2, where the potential atomically dispersed Bi atoms were marked in yellow circles. (d) STEM elemental mapping of Bi_1/CeO_2 catalyst (red, Bi; green, Ce; blue, O)

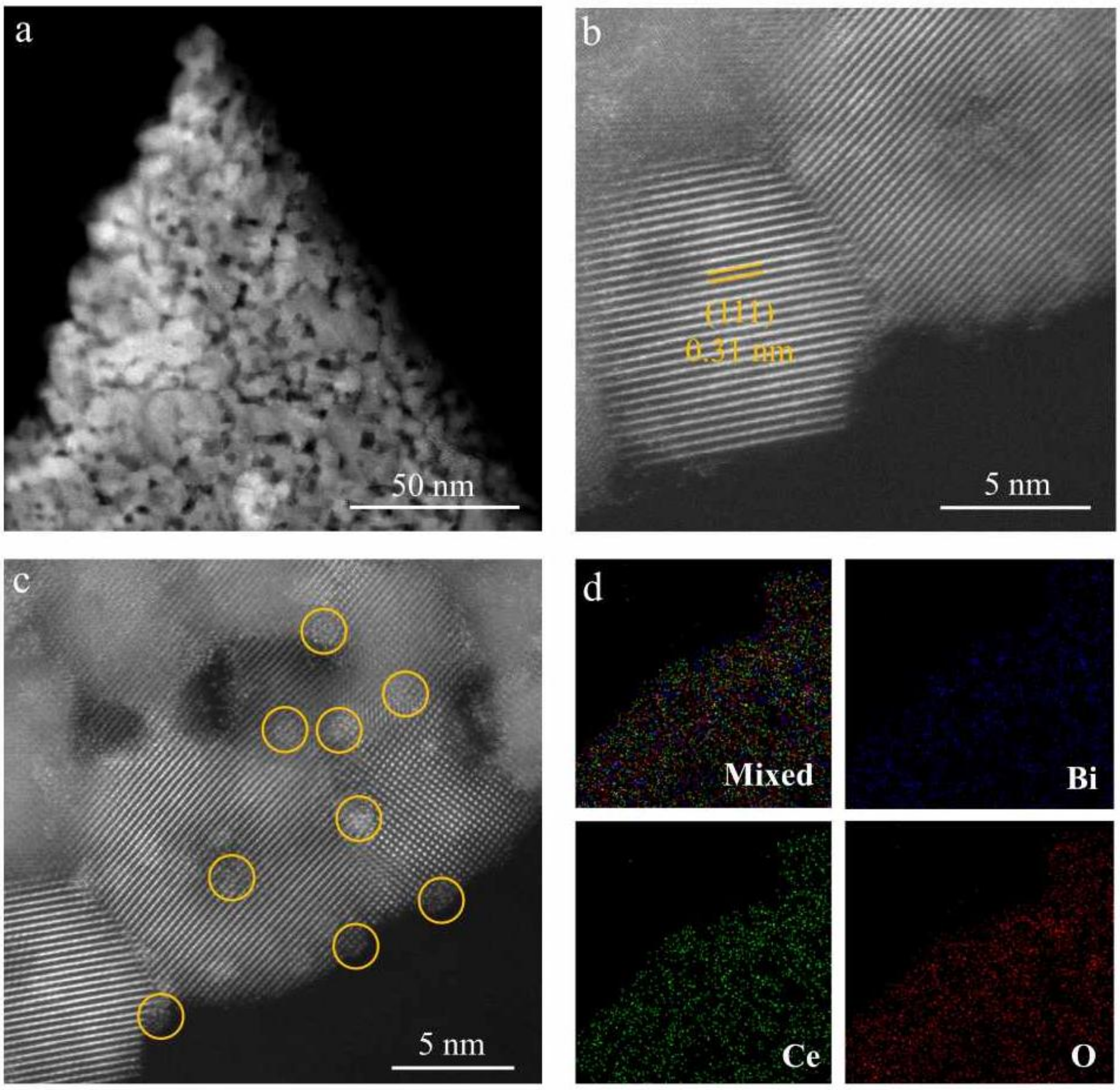

Figure S15. (a) TEM images of Bi_n/CeO_2. (b) HAADF-STEM images of Bi_n/CeO_2 (e) AC-HAADF-STEM image of Bi_n/CeO_2, where the Bi clusters were marked in yellow circles. (d) STEM elemental mapping of Bi_1/CeO_2 catalyst (blue, Bi; green, Ce; red, O)

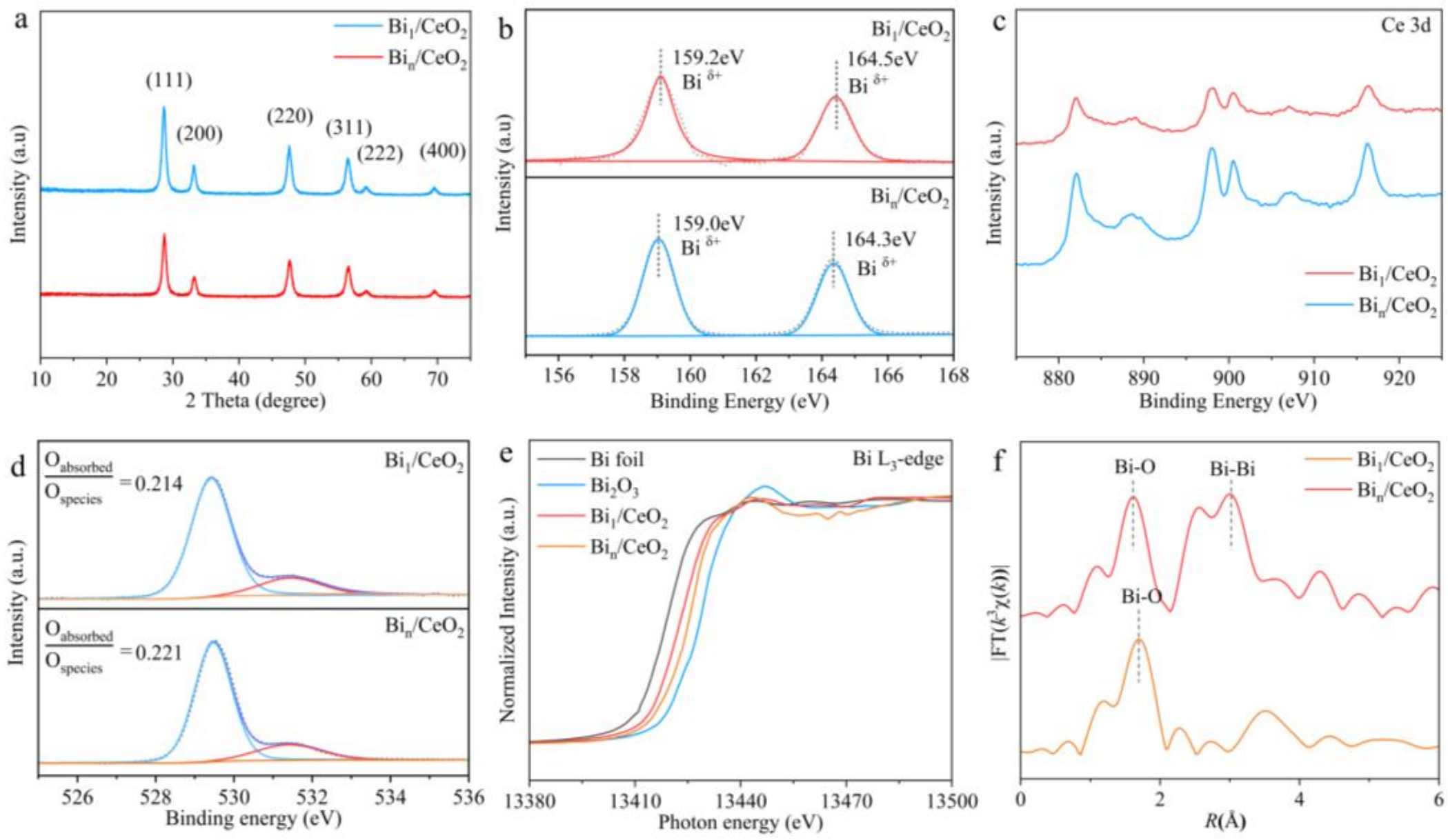

Figure S16. (a) XRD pattern of Bi_1/CeO_2 and Bi_n/CeO_2. (b) Bi 4f XPS spectra for Bi_1/CeO_2 and Bi_n/CeO_2. (c) Ce 3d XPS spectra for Bi_1/CeO_2 and Bi_n/CeO_2. (d) O 1s XPS spectra for Bi_1/CeO_2 and Bi_n/CeO_2. (e) Bi L_3-edge XANES spectra for Bi_1/CeO_2 and Bi_n/CeO_2。(f) Bi L_3-edge EXAFS spectra for Bi_1/CeO_2 and Bi_n/CeO_2.

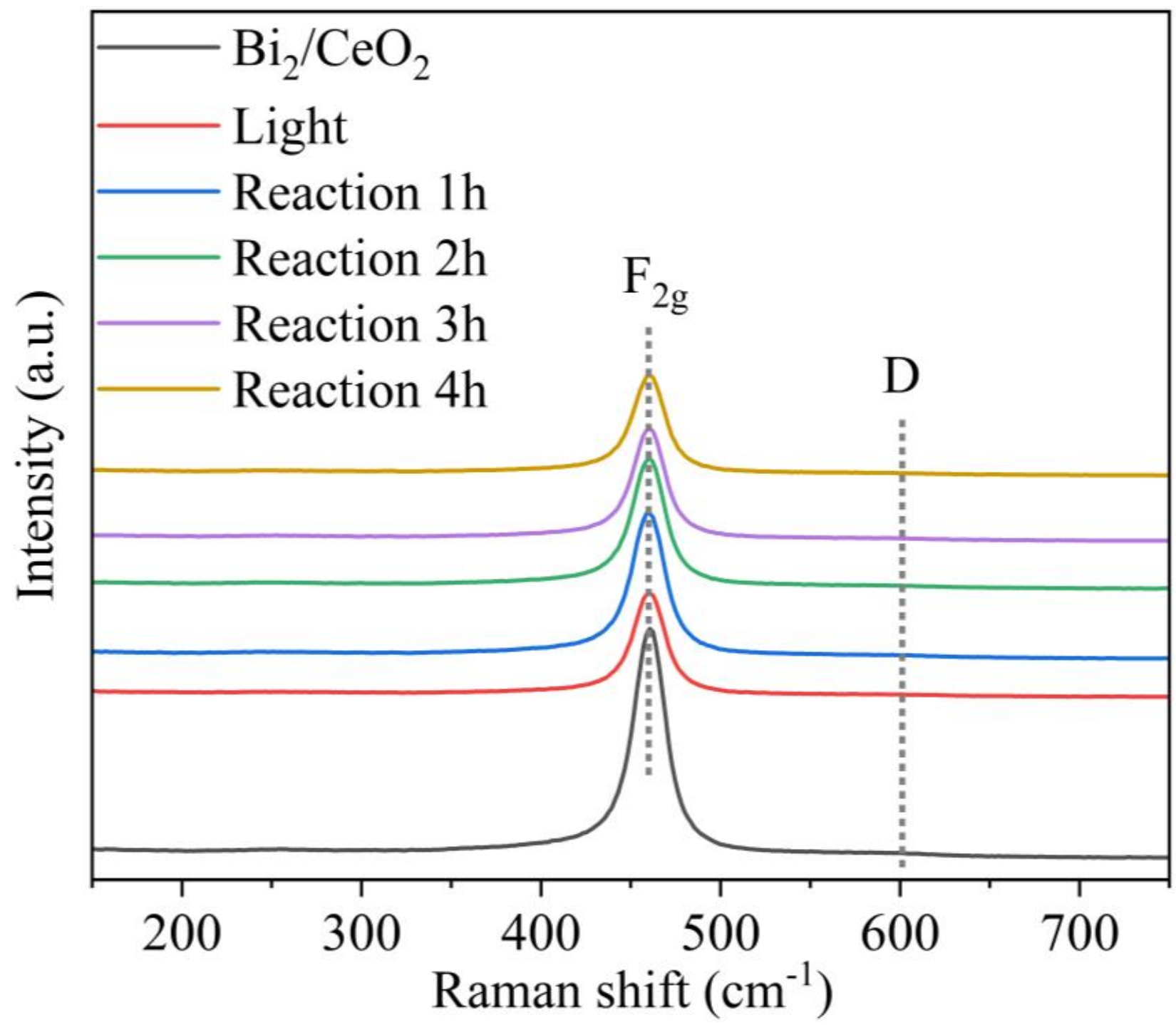

Figure S17. Stability test for Bi_2/CeO_2 based on *in situ* Raman investigations. *In situ* Raman spectra of Bi_2/CeO_2 collected under various conditions: catalyst precursor with reaction conditions under light off (black line), catalyst precursor with reaction conditions under light on (red line), reaction for 1h under light on (blue line), reaction for 2 h under light on (green line), reaction for 3 h under light on (purple line), reaction for 4 h under light on (yellow line). Notably, the F_{2g} peak and D peak of Bi_2/CeO_2 exhibit no shifting under light on and during the reaction time.

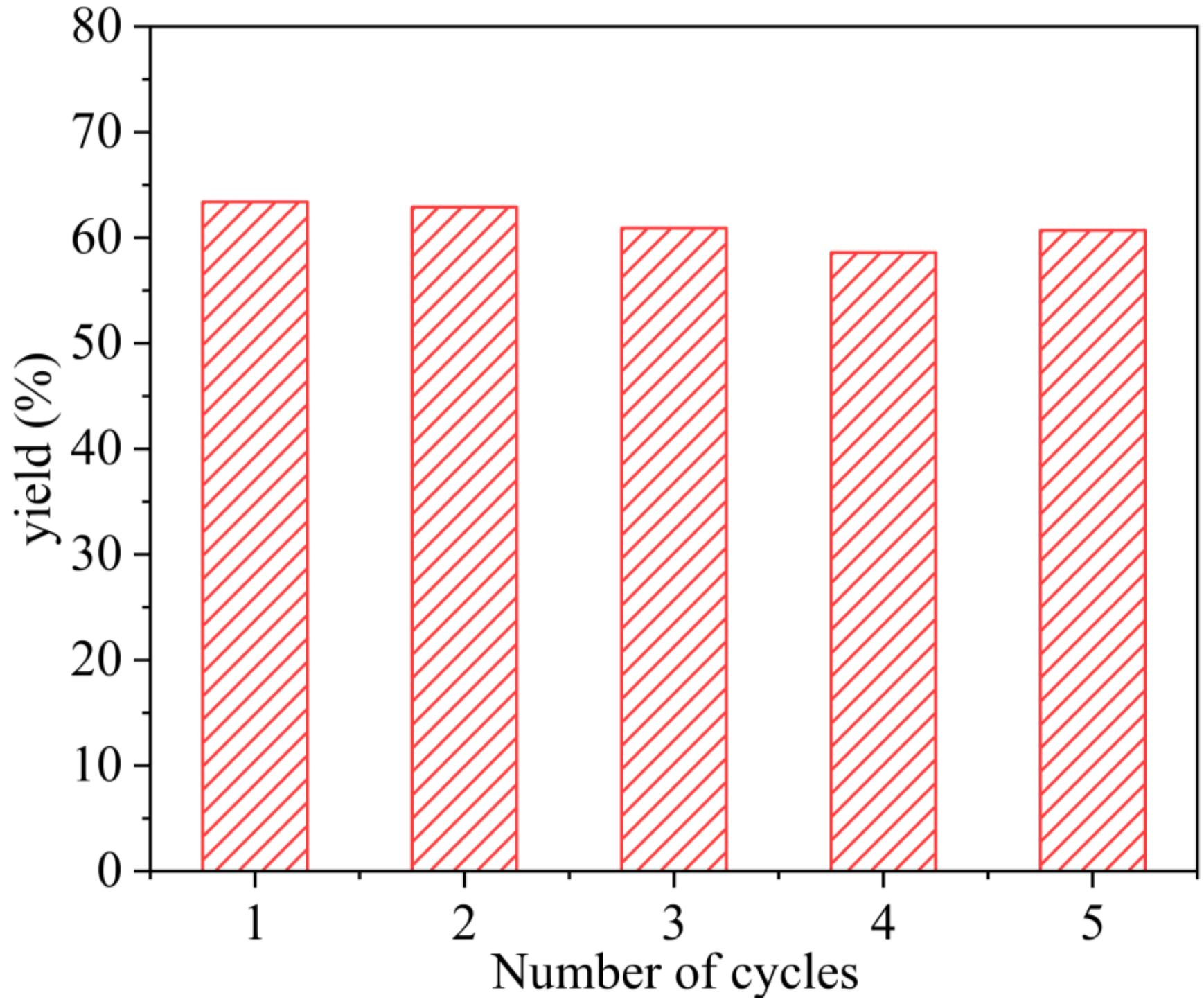

Figure S18. Cycling test of Bi_2/CeO_2 for Photocatalytic styrene oxide cycloaddition with CO_2. Reaction conditions: 10 mmol styrene oxide, 20 mg catalyst, 0.25 mmol TBAB, 1 bar CO_2, 1200 $mW \cdot cm^{-2}$ full-spectrum irradiation, 4 h.

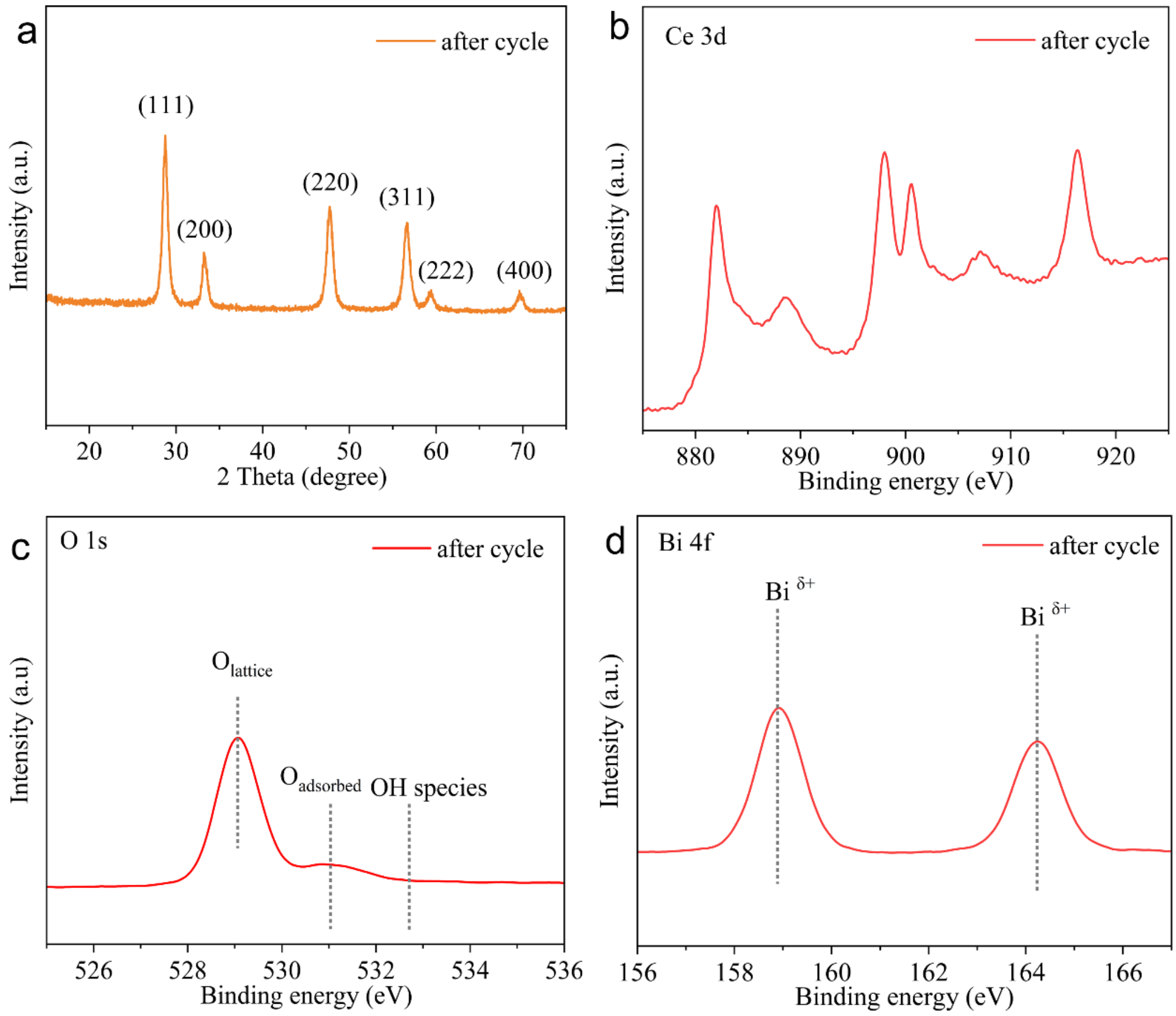

Figure S19. Characterization of the Bi_2/CeO_2 sample after cycling test. (a) XRD pattern. (b) Ce 3d XPS spectra. (c) O 1s XPS spectra. (d) Bi 4f XPS spectra.

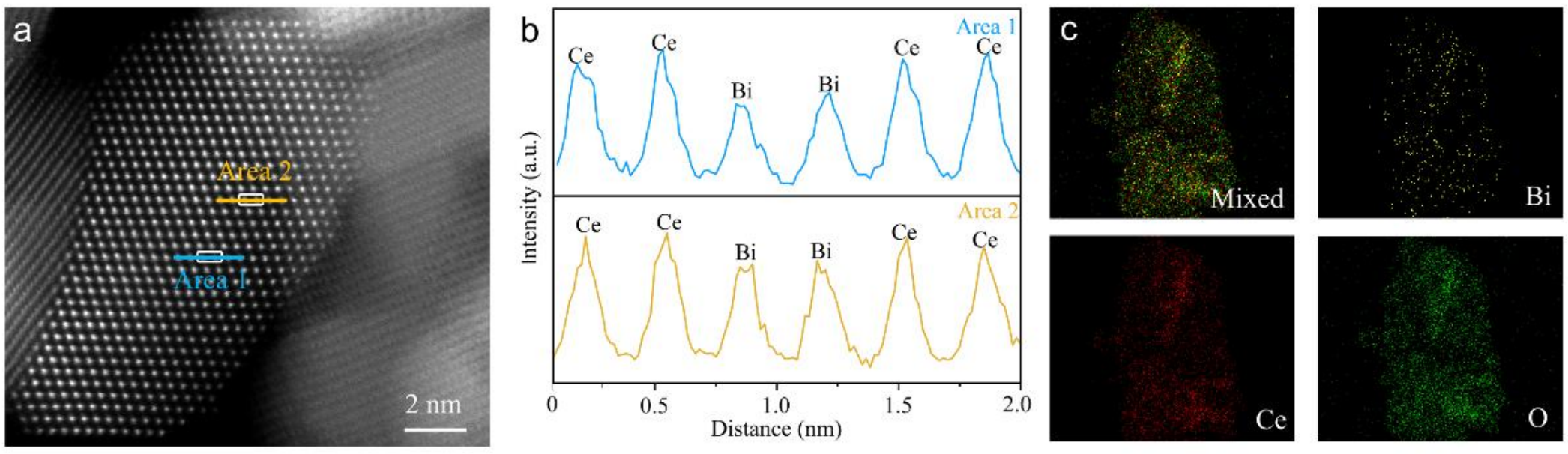

Figure S20. Characterization of the Bi_2/CeO_2 sample after cycling test. (a) AC-HAADF-STEM image and the Bi atom-pairs are highlighted by yellow and blue regions. (b) Brightness intensity of the line profile with the Bi atom-pairs as white regions shown in yellow and blue lines in the AC-HAADF-STEM image. (c) STEM elemental mapping (yellow, Bi; red, Ce; green, O).

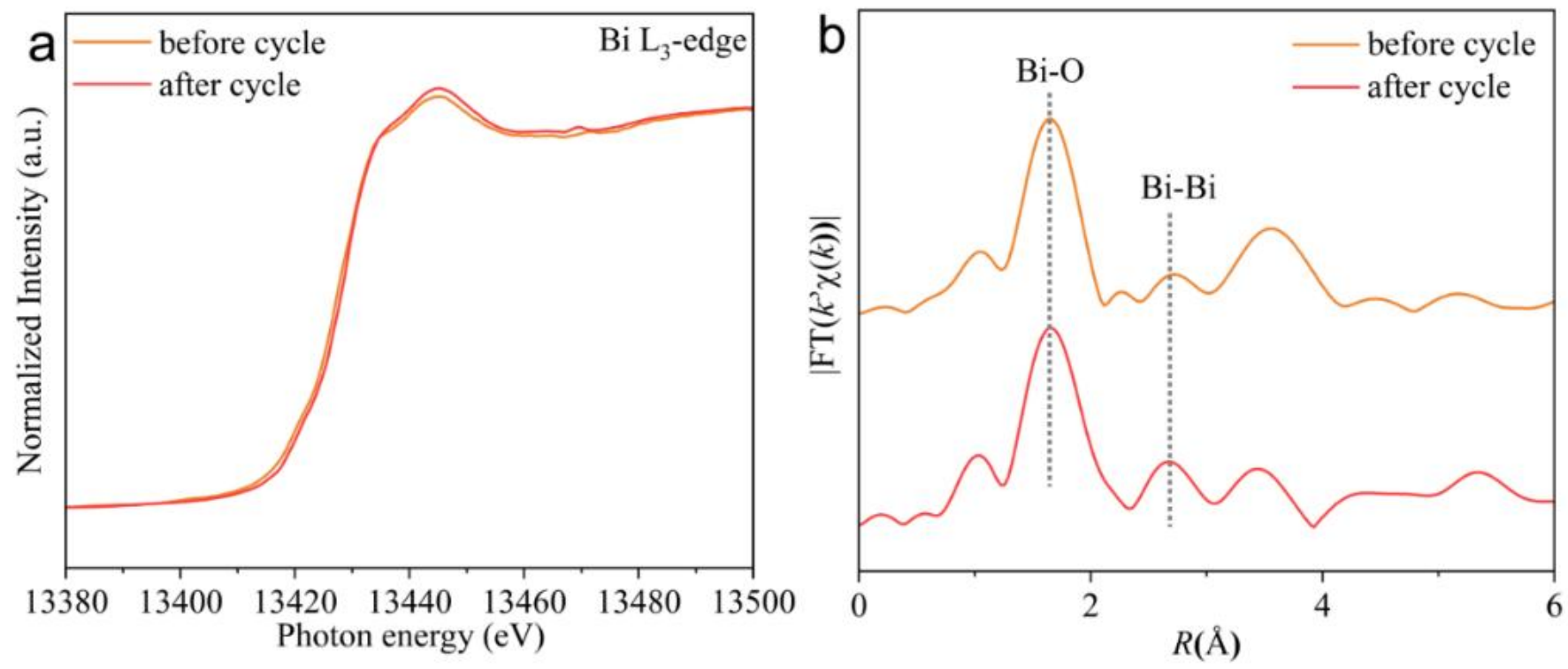

Figure S21. Characterization of the Bi_2/CeO_2 sample after cycling test. (a) Bi-L_3-edge XANES spectra. (b) Bi L_3-edge EXAFS spectra.

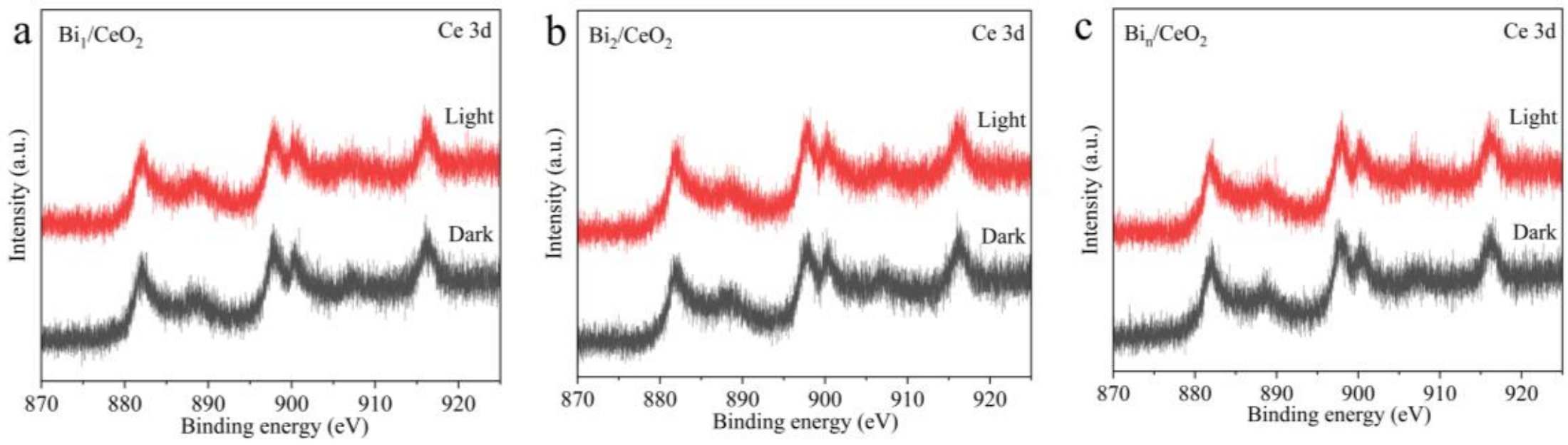

Figure S22. *In situ* XPS Ce 3d spectra of (a) Bi_1/CeO_2, (b) Bi_2/CeO_2 and (c) Bi_n/CeO_2

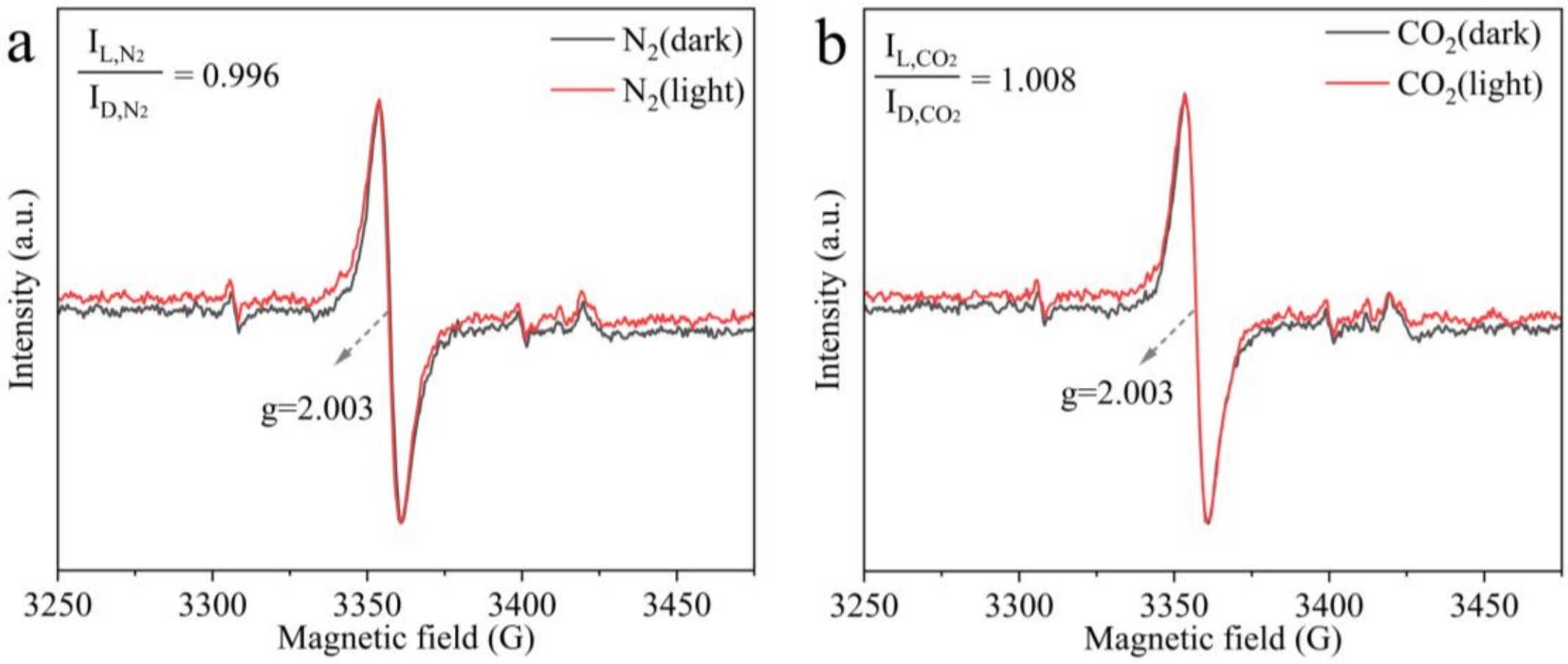

Figure S23. *In situ* low-temperature ESR spectra of Bi_2/CeO_2 before and after the introduction of light irradiation with different condition of atmosphere: (a) in N_2 and (b) in CO_2.

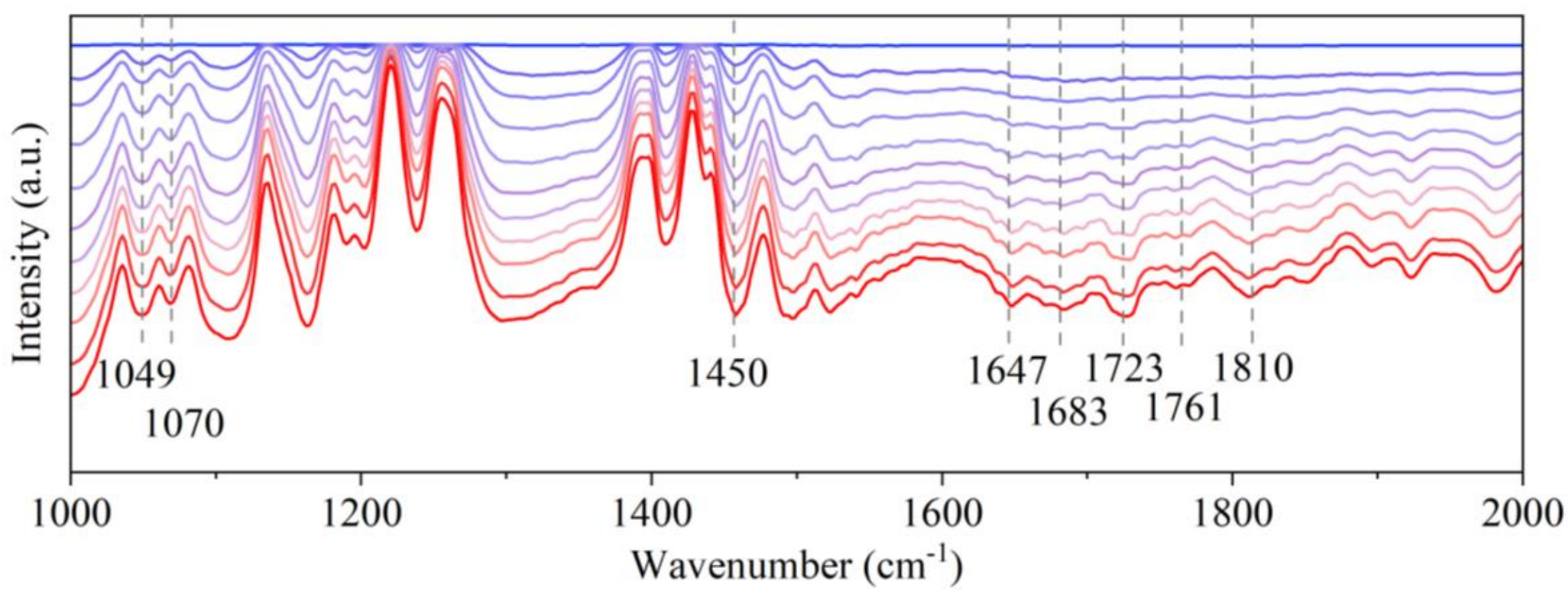

Figure S24. *In situ* DRIFTS of Bi_2/CeO_2 for the photocatalytic CO_2 cycloaddition with styrene oxide under simulated experimental condition.

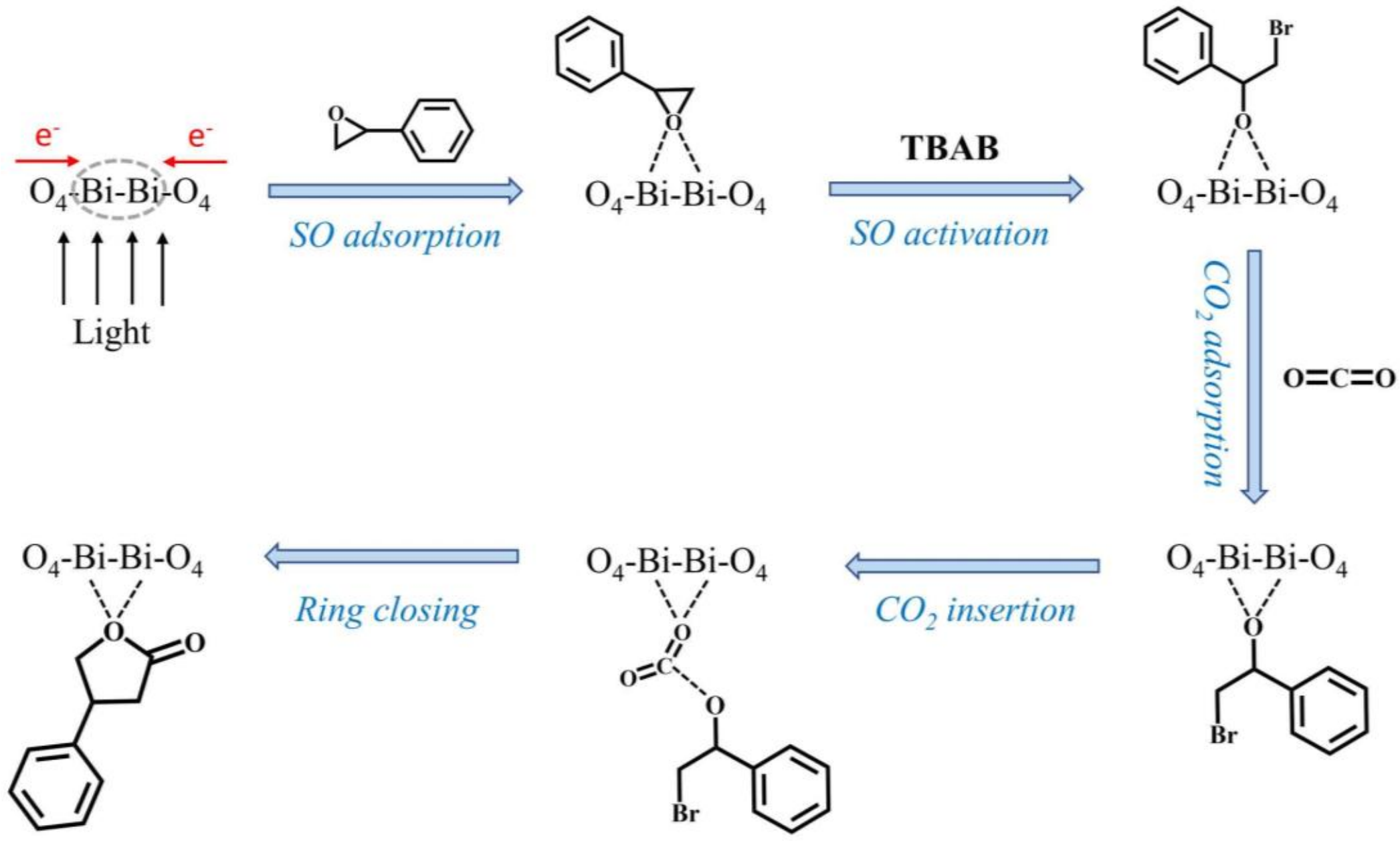

Figure S25. Proposed mechanism for photocatalytic styrene oxide cycloaddition catalyzed by Bi_2/CeO_2.

Table S1. Inductively coupled plasma optical emission spectroscopy (ICP-OES) characterization of Bi_2/CeO_2

Sample	Bi loading	Average Bi loading
**Bi_2/CeO_2-1**	1.49%	
**Bi_2/CeO_2-2**	1.50%	1.47%
**Bi_2/CeO_2-3**	1.43%	

Table S2. EXAFS fitting parameters at the Bi L_3-edge for various samples.

**Condition**	**Pair**	***[a]CN***	***[b]R* (Å)**	**$^c\sigma^2$ (10^{-3}Å^2)**	**$^d\Delta E_0$ (eV)**	R factor
Bi foil	Bi-Bi	3	3.10	-	-	-
	Bi-Bi	3	3.59	-	-	
Bi_1/CeO_2	Bi-O	3.0±0.7	2.19±0.01	7.0±2.5	-4.4±0.9	0.02
Bi_2/CeO_2	Bi-O	3.7±0.5	2.16±0.01	6.9±0.8	-3.3±0.7	0.02
	Bi-Bi	1.1±0.2	3.05±0.01	8.0±1.0	-9.9±1.9	
Bi_2/CeO_2-used	Bi-O	3.7±0.8	2.16±0.02	6.9±0.7	-3.6±0.7	0.02
	Bi-Bi	1.0±0.2	3.06±0.01	8.9±0.9	-9.6±1.8	

[a]CN is the coordination number for the absorber-backscatter pair, [b]R is the average absorber-backscatter distance, $^c\sigma^2$ is the Debye-Waller factor, and $^d\Delta E_0$ is the inner potential correction and R factor indicates the goodness of the fit.

Table S3. Comparisons of recent researches for styrene oxide cycloaddition with CO_2.

Catalyst	Reaction Conditions	Solvent	Co-catalyst	SO reaction rate (mmol g_{cat}^{-1} h^{-1})	Ref.
**Bi_2/CeO_2**	1.2W·cm^{-2} Xe lamp	-	TBAB	81.25	*This work*
**13.3 Ti-CNO**	2W·cm^{-2} Xe lamp	-	TBAB	62.5	Ref[17]
**ZNC-800**	1W·cm^{-2} Xe lamp	-	TBAB	43.8	Ref[18]
**$Ti_{18}Bi_4$**	0.4W·cm^{-2} Xe lamp	-	TBAB	28.6	Ref[19]
**Ni-BNCNTs @HMPs-NH_2**	0.4W·cm^{-2} Xe lamp	-	TBAB	10.5	Ref[20]
**COP-222**	100 °C	-	TBAB	6.9	Ref[21]
**Ir-WO_3**	40 °C	-	TBAB	6.7	Ref[22]
**PILs@Al-O-C**	110 °C	-		2.76	Ref[23]
**Al-N-C**	0.4W·cm^{-2} Xe lamp	2ml DMF	TBAB	0.97	Ref[24]
**HPC-800**	0.3W·cm^{-2} Xe lamp	3ml DMF	TBAB	0.07	Ref[25]

References

[1] S. Shang, L. Li, H. Wang, X. Zhang, Y. Xie, *Nano Lett.* **2023,** 16, 7650–7657.

[2] B. Ravel, M. Newville, *J. Synchrotron Radiat.* **2005**, *12*, 537-541.

[3] M. Newville, *J. Synchrotron Radiat.* **2001**, *8*, 322-324.

[4] G. Kresse, J. Furthmuller, *Computational Comput. Mater. Sci.* **1996**, *6*, 15-50.

[5] J. P. Perdew, K. Burke, M. Ernzerhof, *Phys. Rev. Lett.* **1996**, *77*, 3865-3868.

[6] G. Kresse, D. Joubert, *Phys. Rev. B.* **1999**, *59*, 1758-1775.

[7] S. L. Dudarev, G. A. Botton, S. Y. Savrasov, C. J. Humphreys, A. P. Sutton, *P Phys. Rev. B.* **1998**, *57*, 1505-1509.

[8] aJ. L. F. Da Silva, M. V. Ganduglia-Pirovano, J. Sauer, V. Bayer, G. Kresse, *P Phys. Rev. B.* **2007**, *75*; bC. Loschen, J. Carrasco, K. M. Neyman, F. Illas, *P Phys. Rev. B.* **2007**, *75*.

[9] a) Y.-Q. Su, I. A. W. Filot, J.-X. Liu, I. Tranca, E. J. M. Hensen, *Chem Mater.* **2016**, *28*, 5652-5658; b) M. Capdevila-Cortada, M. Garcia-Melchor, N. Lopez, *J. Catal.* **2015**, *327*, 58-64; c) R. Farra, M. Garcia-Melchor, M. Eichelbaum, M. Hashagen, W. Frandsen, J. Allan, F. Girgsdies, L. Szentmiklosi, N. Lopez, D. Teschner, *ACS Catal.* **2013**, *3*, 2256-2268.

[10] a) J. J. Rehr, R. C. Albers, *Rev. Mod. Phys.* **2000**, *72*, 621-654; b) Y. Joly, *Journal De Physique Iv.* **1997**, *7*, 111-115; c) O. Bunau, Y. Joly, *J Phys-Condents Mat.* **2009**, *21*.

[11] G. Henkelman, B. P. Uberuaga, H. Jónsson, *J. Chem. Phys.* **2000**, *113*, 9901-9904.

[12] Y. Wang, H. Liu, Q. Shi, Z. Miao, H. Duan, Y. Wang, H. Rong, J. Zhang, *Angew. Chem. Int. Ed.* **2024**, *63*, e202404911.

[13] Y. Liu, Y. Chen, Y. Liu, Z. Chen, H. Yang, Z. Yue, Q. Fang, Y. Zhi, S. Shan, *J. Catal.* **2022**, *407*, 65-76.

[14] C. Liu, H. Niu, D. Wang, C. Gao, A. Said, Y. Liu, G. Wang, C.-H. Tung, Y. Wang, *ACS Catal.* **2022**, *12*, 8202-8213.

[15] Y. Guo, W. Chen, L. Feng, Y. Fan, J. Liang, X. Wang, X. Zhang, *J. Mater. Chem. A.* **2022**, *10*, 12418-12428.

[16] S. Subramanian, J. Oppenheim, D. Kim, T. S. Nguyen, W. M. H. Silo, B. Kim, W. A. Goddard, C. T. Yavuz, *Chem.* **2019**, *5*, 3232-3242.

[17] J. Xu, H. Xu, A. Dong, H. Zhang, Y. Zhou, H. Dong, B. Tang, Y. Liu, L. Zhang, X. Liu, J. Luo, L. Bie, S. Dai, Y. Wang, X. Sun, Y. Li, *Adv. Mater.* **2022**, *34*, 2206991.

[18] H. Peng, Q. Zhang, Y. Wang, H. Gao, N. Zhang, J. Zhou, L. Zhang, Q. Yang, Q. Yang, Z. Lu, *Appl. Catal. B: Environ.* **2022**, *313*, 121463.

[19] Q. Yang, H. Peng, Q. Zhang, X. Qian, X. Chen, X. Tang, S. Dai, J. Zhao, K. Jiang, Q. Yang, J. Sun, L. Zhang, N. Zhang, H. Gao, Z. Lu, L. Chen, *Adv. Mater.* **2021**, *33*, 2103186

[20] Q. Yang, C.-C. Yang, C.-H. Lin, H.-L. Jiang, *Angew. Chem. Int. Ed.* **2019**, *58*, 3511-3515.
